# Supplementary figures and images for: CRMP4-mediated fornix development involves Semaphorin-3E signaling pathway
Source: eLife. 2021 Dec 3;10:e70361. doi: 10.7554/eLife.70361 (PMC8683083; doi:10.7554/eLife.70361)

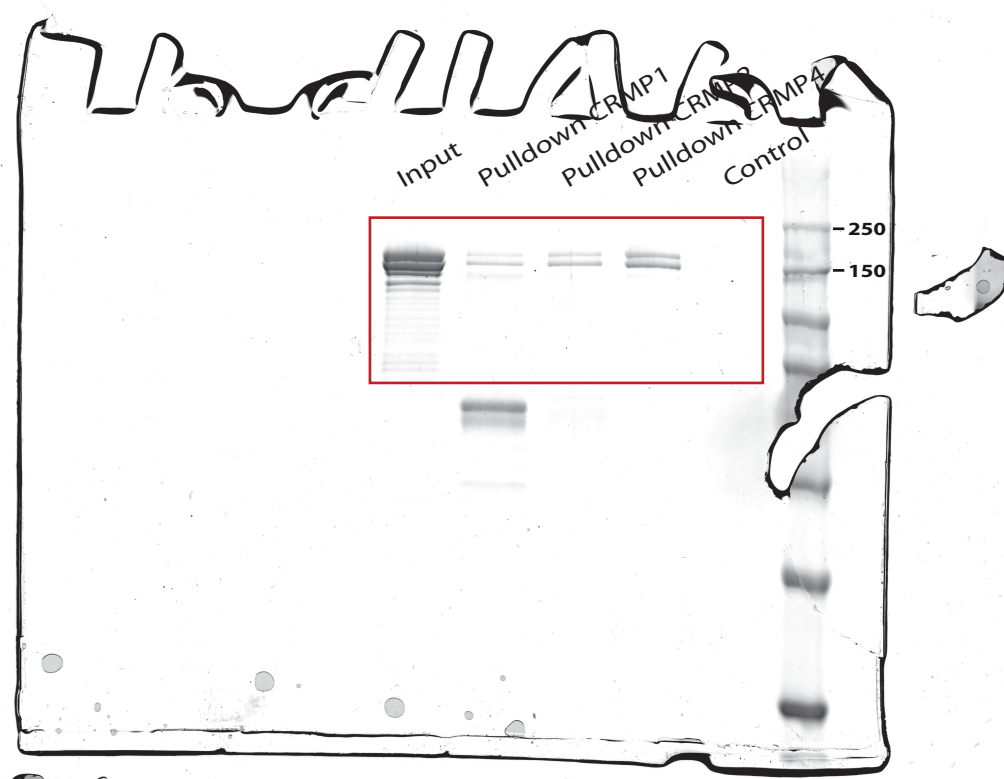

Figure 1-figure supplement 1

Supplement: Figure 1—source data 1. [file elife-70361-fig1-data1.zip › Figure 1-Source Data 1/Figure 1B uncroppped gel with relevant bands .pdf]

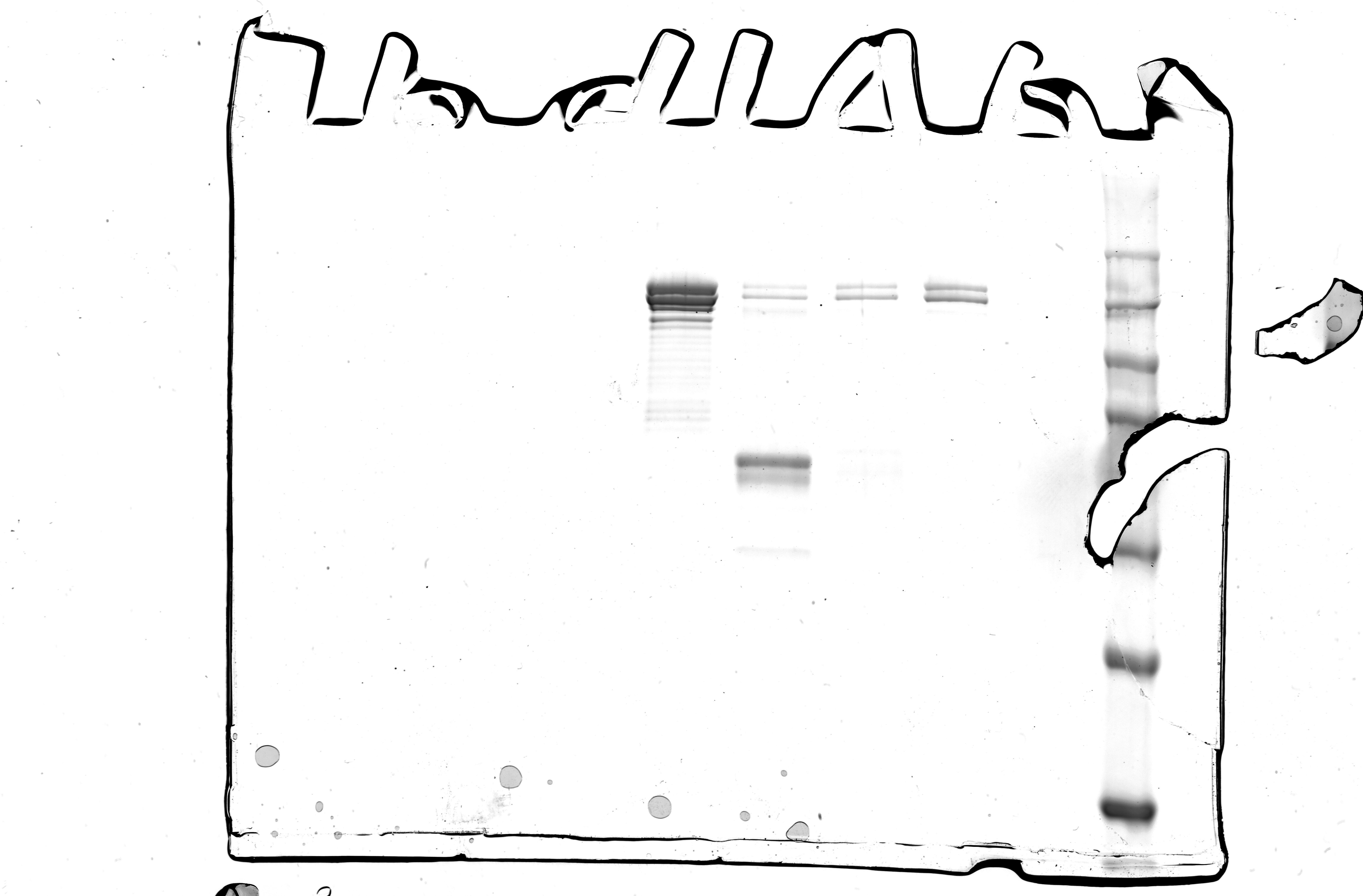

Supplement: Figure 1—source data 1. [file elife-70361-fig1-data1.zip › Figure 1-Source Data 1/Figure 1B full raw unedited.tif]

Figure 1A

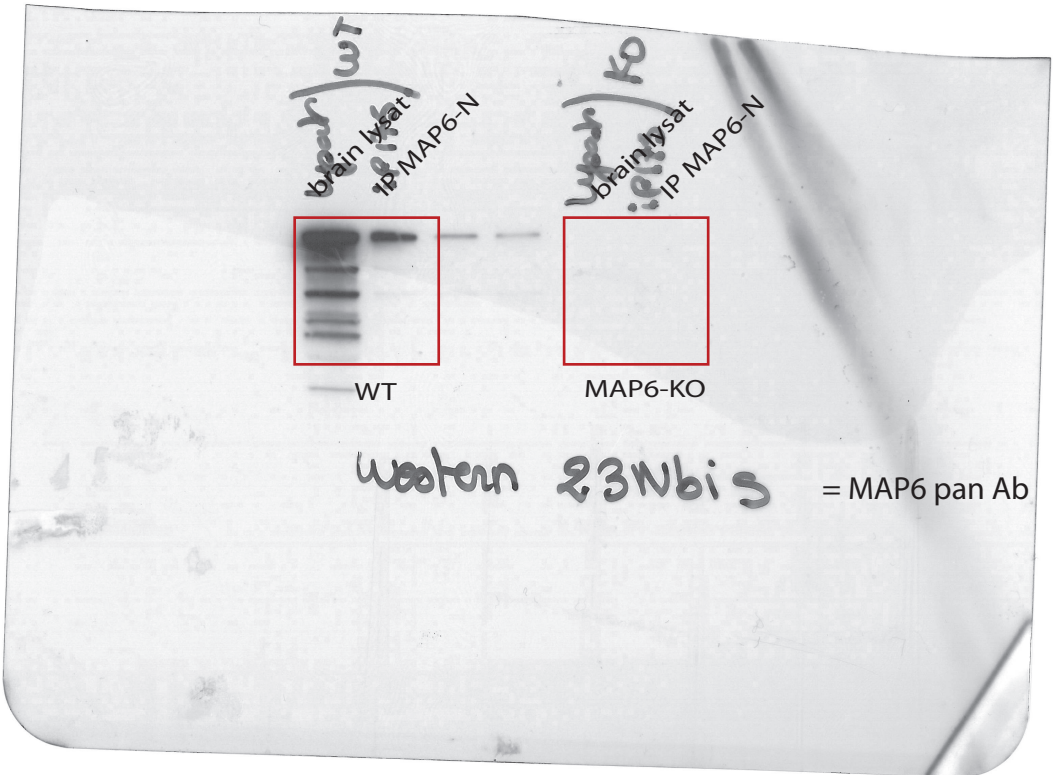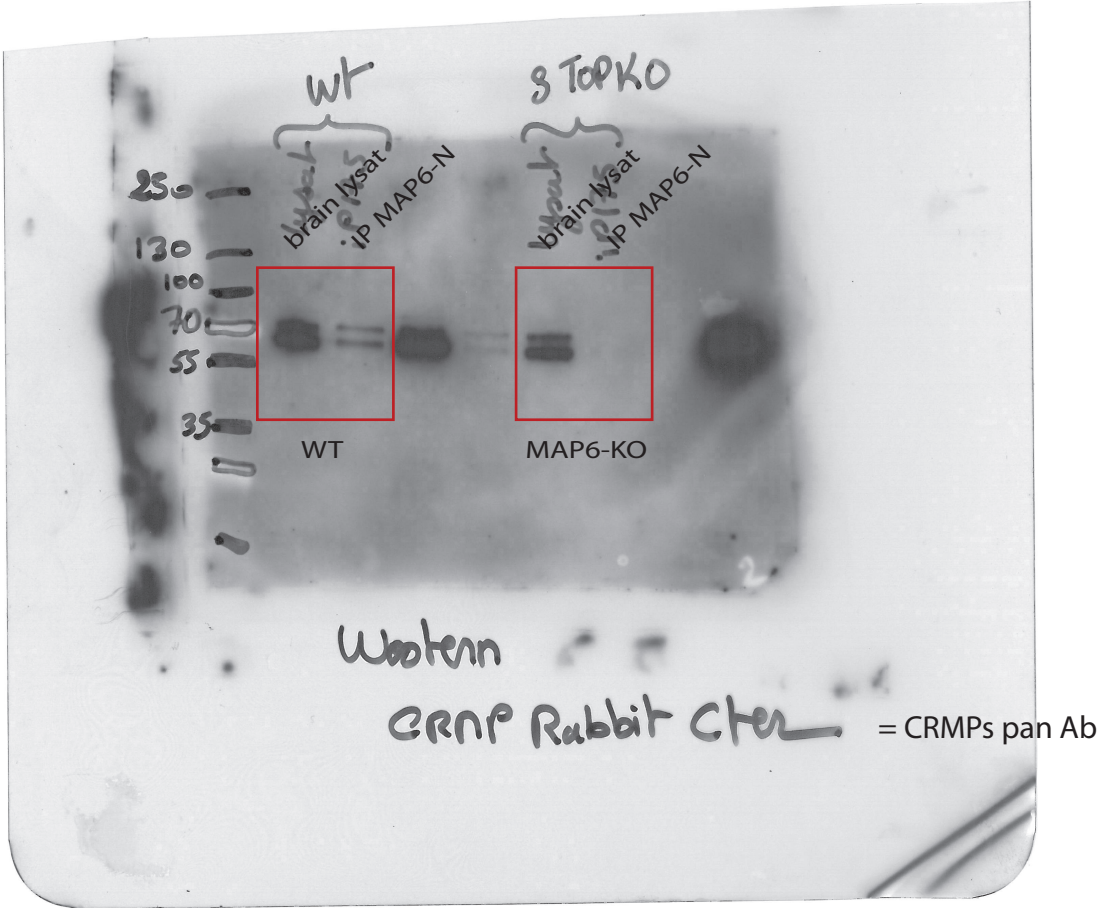

Supplement: Figure 1—source data 1. [file elife-70361-fig1-data1.zip › Figure 1-Source Data 1/Figure 1A uncroppped blot with relevant bands .pdf]

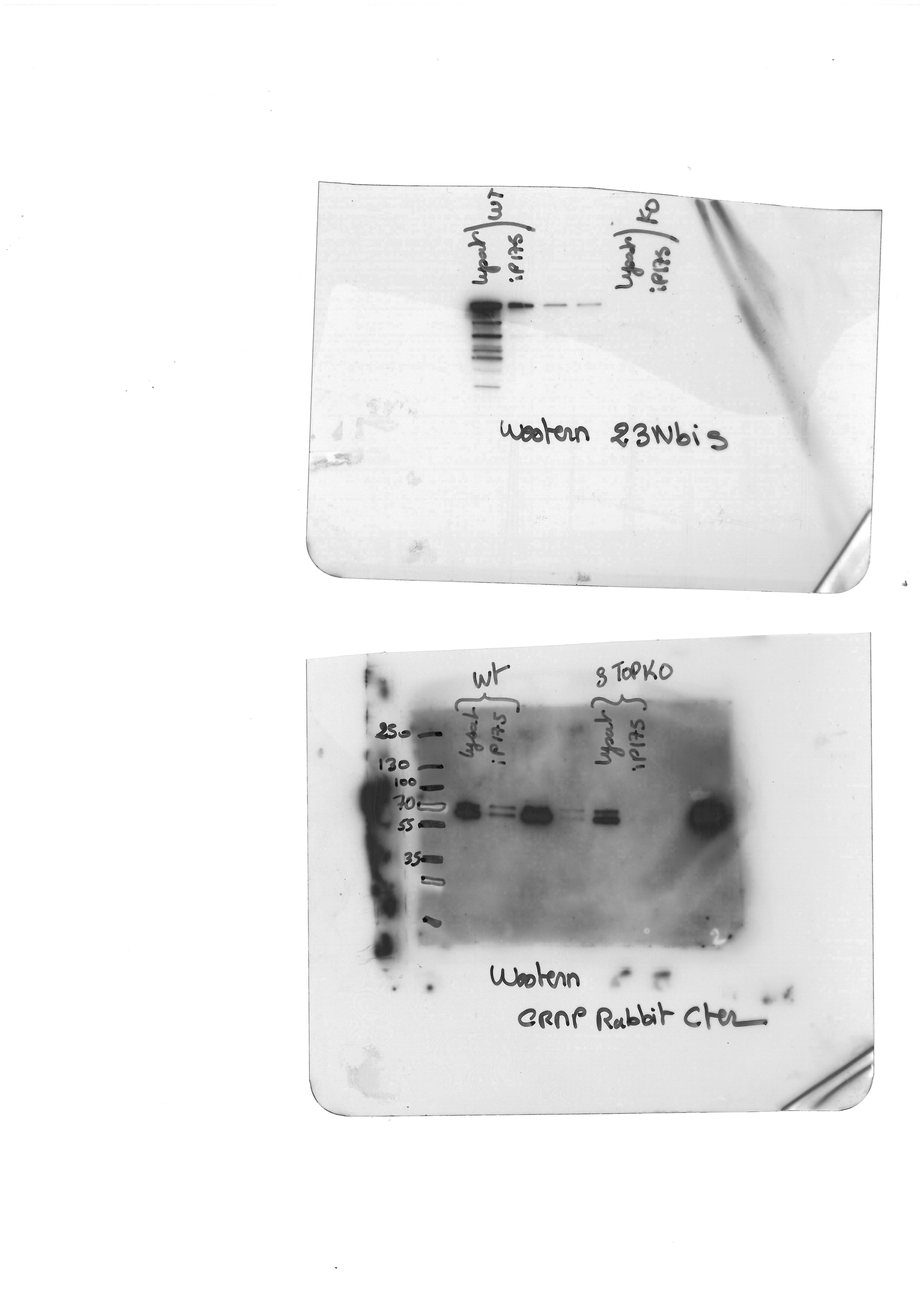

Supplement: Figure 1—source data 1. [file elife-70361-fig1-data1.zip › Figure 1-Source Data 1/Figure 1A full raw unedited.tif]

FIGURE 2A

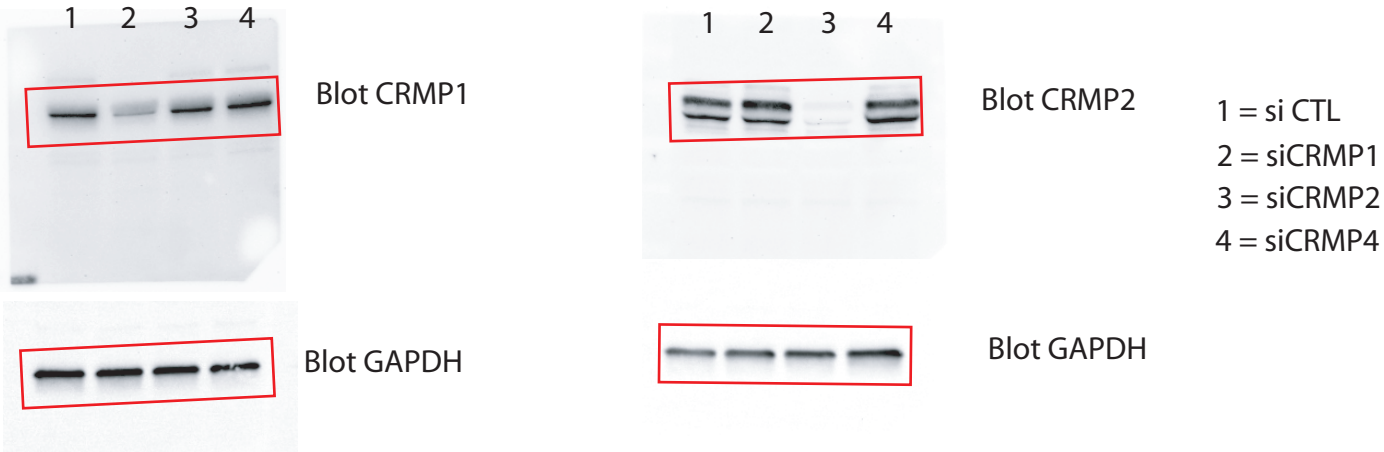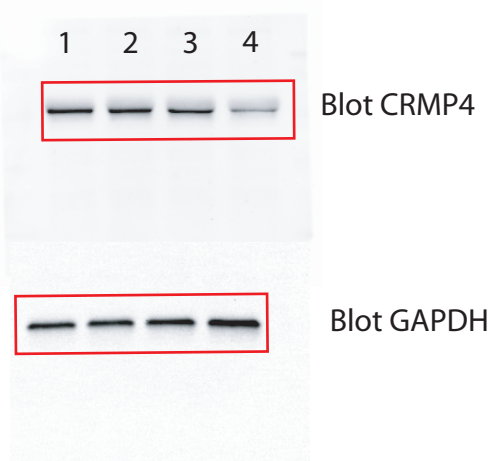

Supplement: Figure 2—source data 1. [file elife-70361-fig2-data1.zip › Figure 2-Source Data 1/Figure 2A uncroppped blot CRMP1 and CRMP4 with relevant bands.pdf]

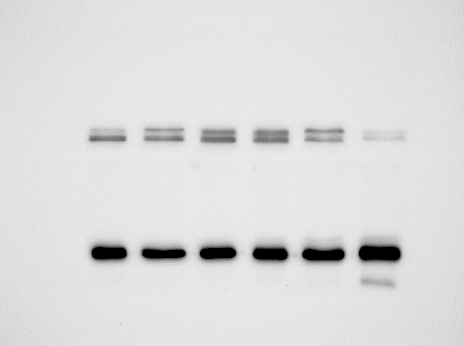

Supplement: Figure 3—figure supplement 1—source data 1. [file elife-70361-fig3-figsupp1-data1.zip › Figure 3A-figure suplement 1-Source Data 1/Figure 3A full raw unedited.tif]

Figure 3A supplement 1

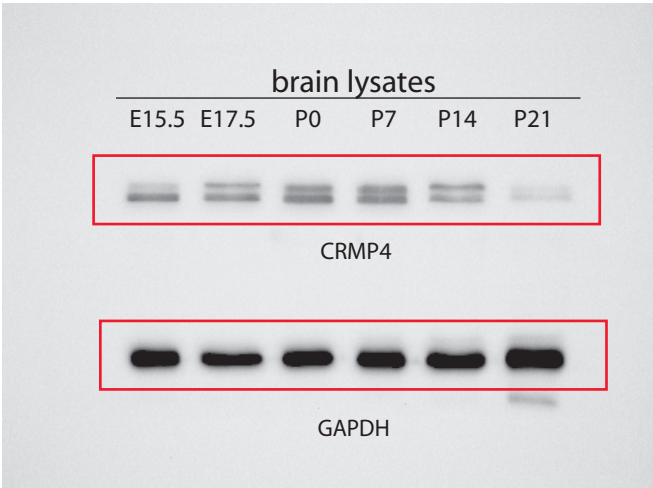

Supplement: Figure 3—figure supplement 1—source data 1. [file elife-70361-fig3-figsupp1-data1.zip › Figure 3A-figure suplement 1-Source Data 1/Figure 3A supplement 1 uncroppped blot with relevant bands .pdf]

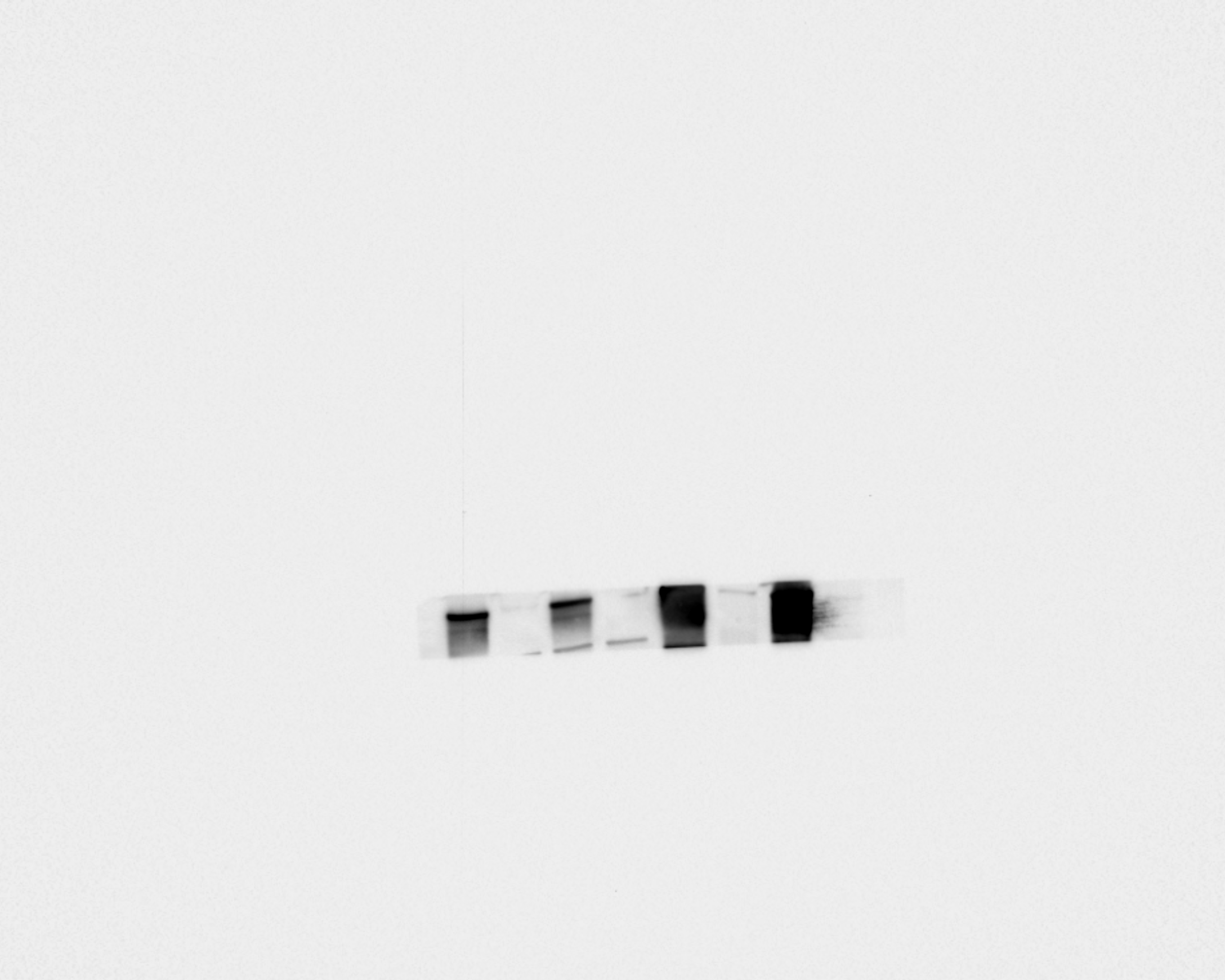

Supplement: Figure 7—source data 1. [file elife-70361-fig7-data1.zip › Figure 7-Source Data 1/Figure 7 full raw unedited/Figure 7C CRMP4.tif]

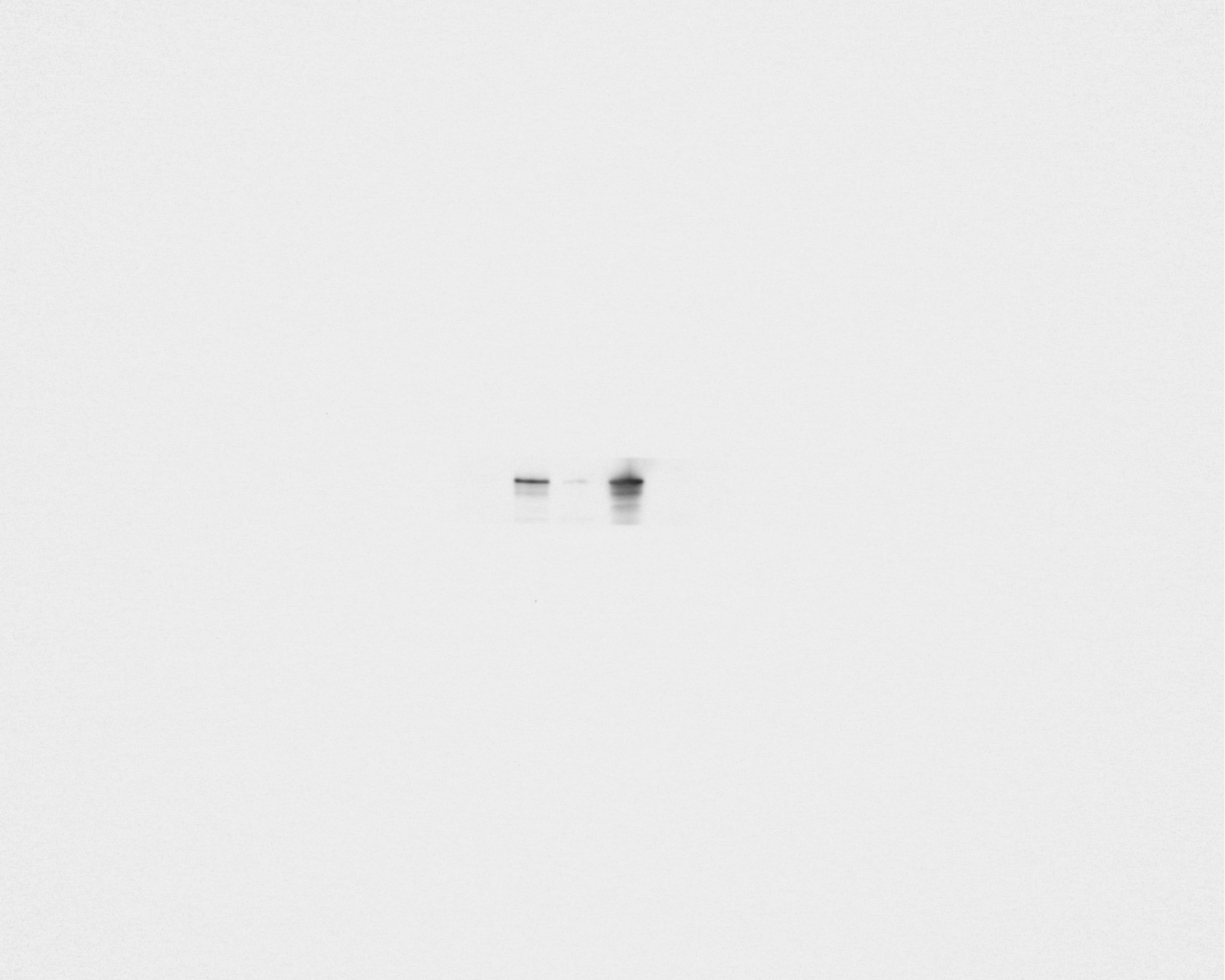

Supplement: Figure 7—source data 1. [file elife-70361-fig7-data1.zip › Figure 7-Source Data 1/Figure 7 full raw unedited/Figure 7B CRMP4.tif]

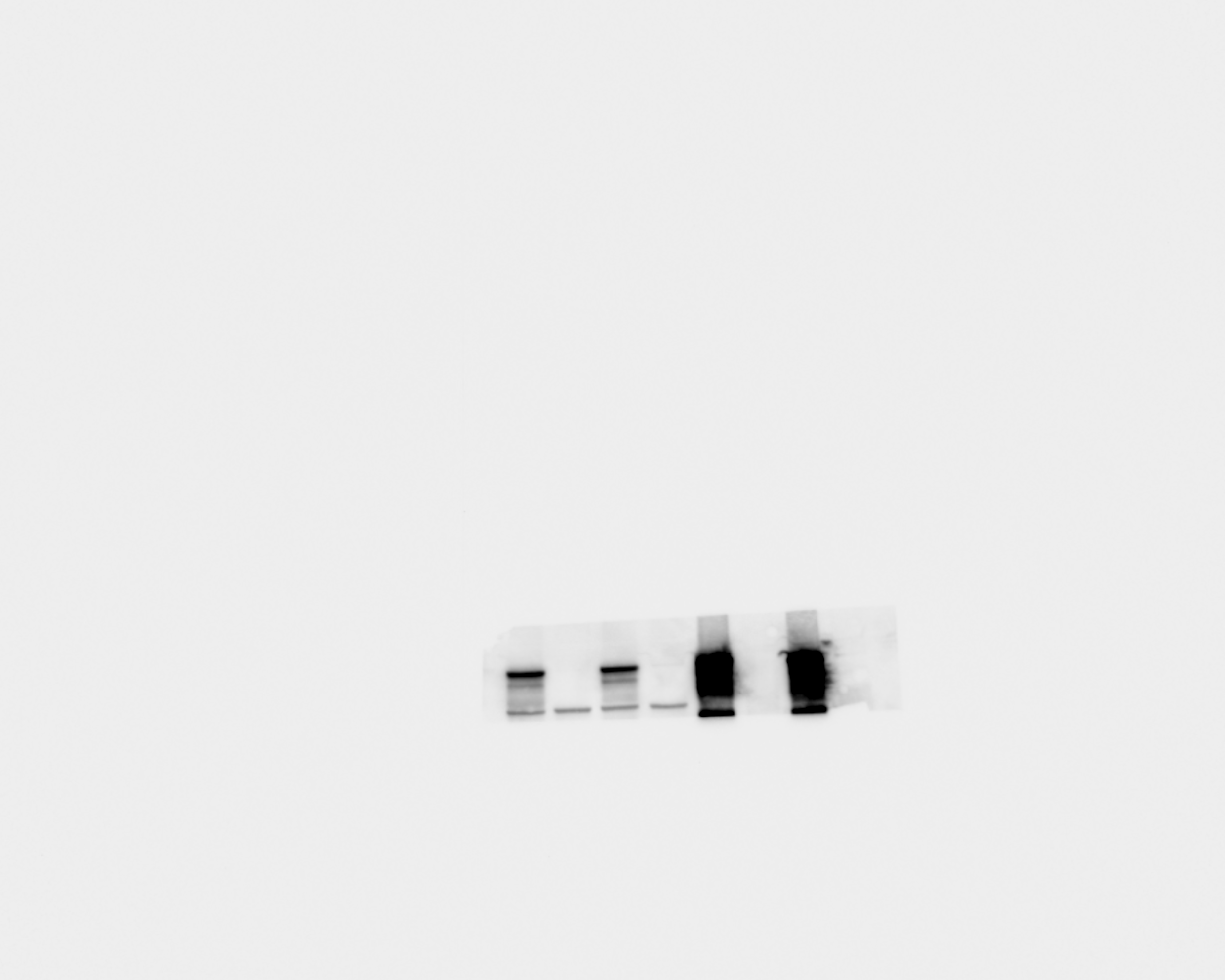

Supplement: Figure 7—source data 1. [file elife-70361-fig7-data1.zip › Figure 7-Source Data 1/Figure 7 full raw unedited/Figure 7D CRMP4.tif]

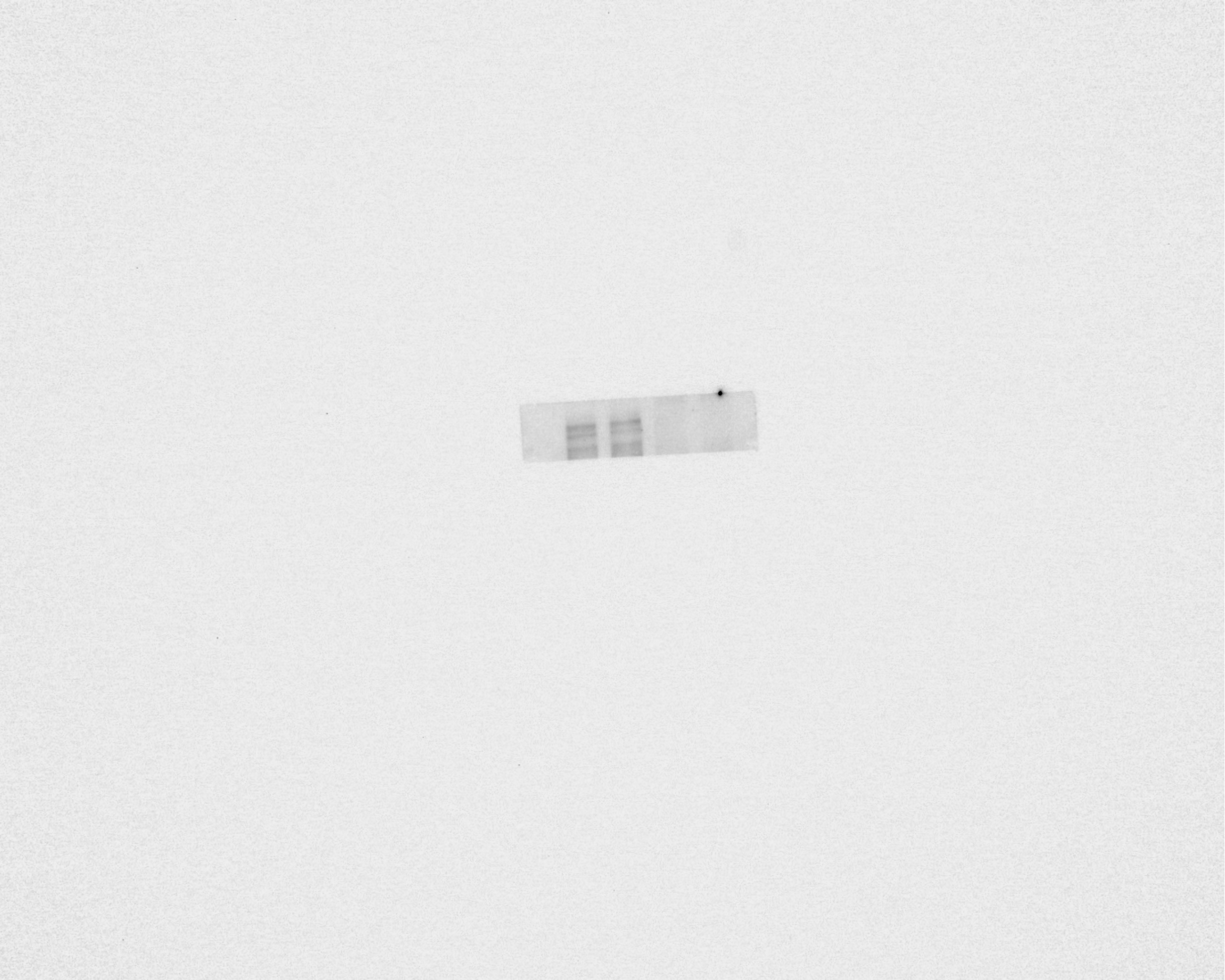

Supplement: Figure 7—source data 1. [file elife-70361-fig7-data1.zip › Figure 7-Source Data 1/Figure 7 full raw unedited/Figure 7B VEGRF2.tif]

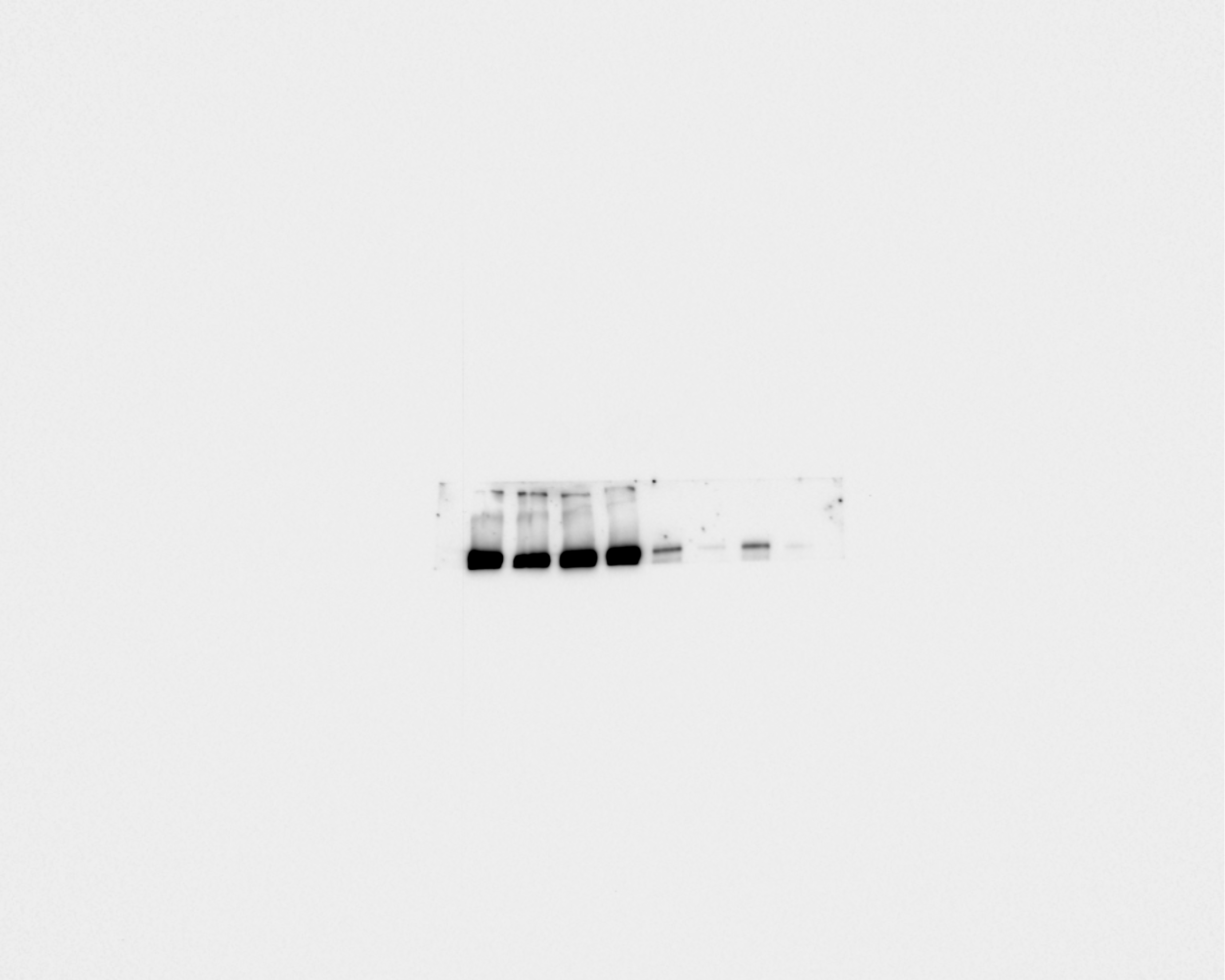

Supplement: Figure 7—source data 1. [file elife-70361-fig7-data1.zip › Figure 7-Source Data 1/Figure 7 full raw unedited/Figure 7A PlxD1.tif]

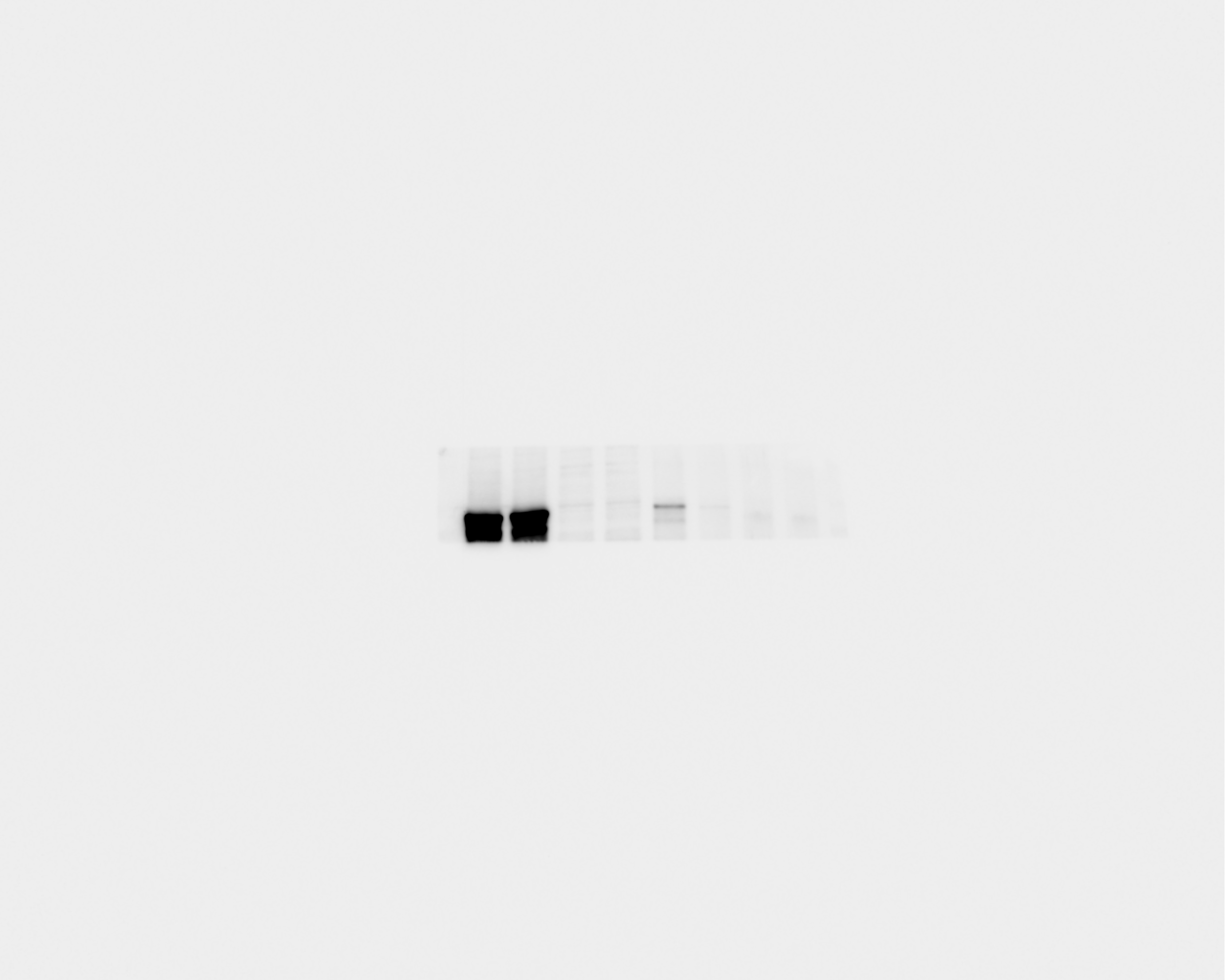

Supplement: Figure 7—source data 1. [file elife-70361-fig7-data1.zip › Figure 7-Source Data 1/Figure 7 full raw unedited/Figure 7A CRMP4.tif]

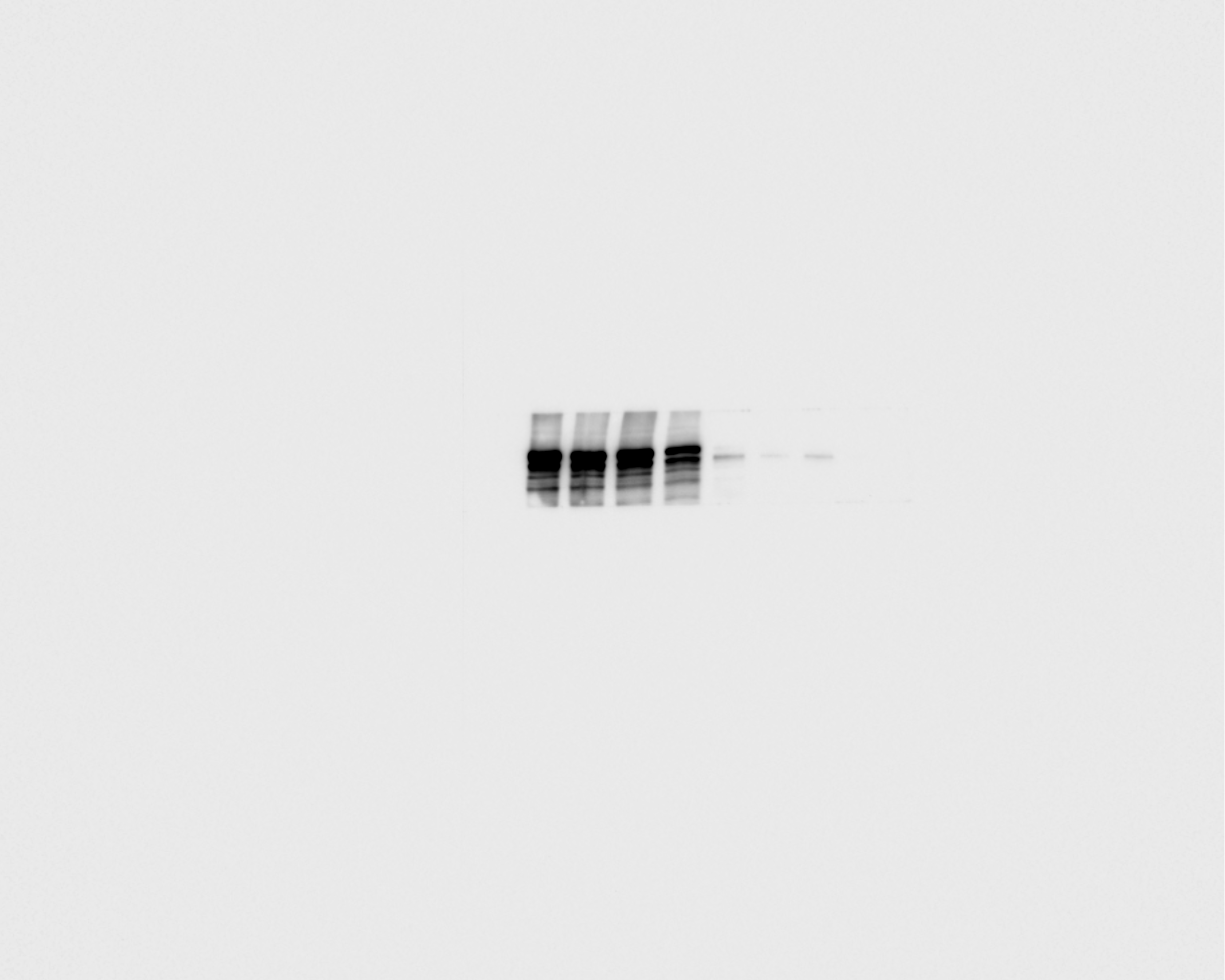

Supplement: Figure 7—source data 1. [file elife-70361-fig7-data1.zip › Figure 7-Source Data 1/Figure 7 full raw unedited/Figure 7A NRP1.tif]

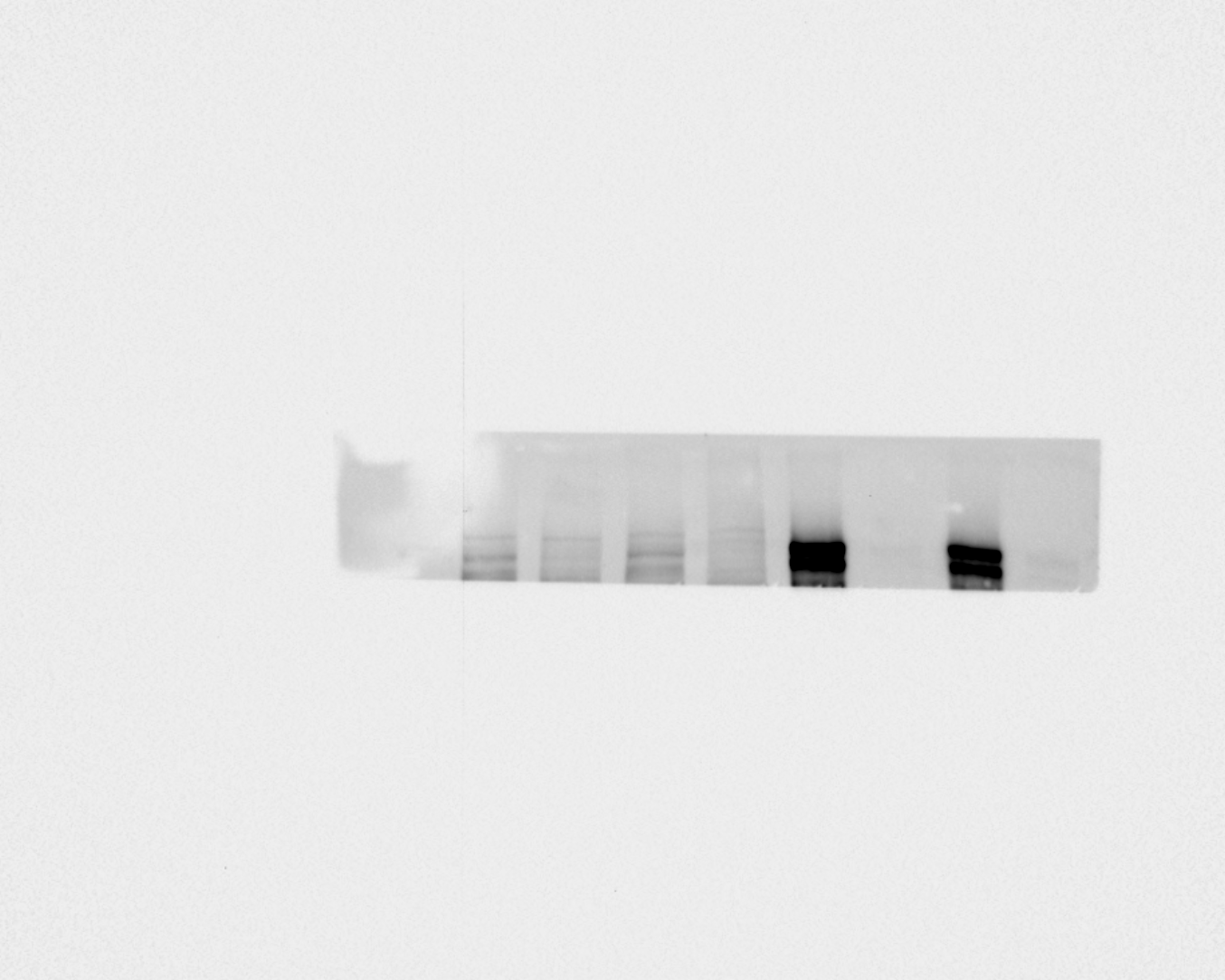

Supplement: Figure 7—source data 1. [file elife-70361-fig7-data1.zip › Figure 7-Source Data 1/Figure 7 full raw unedited/Figure 7A VEGFR2.tif]

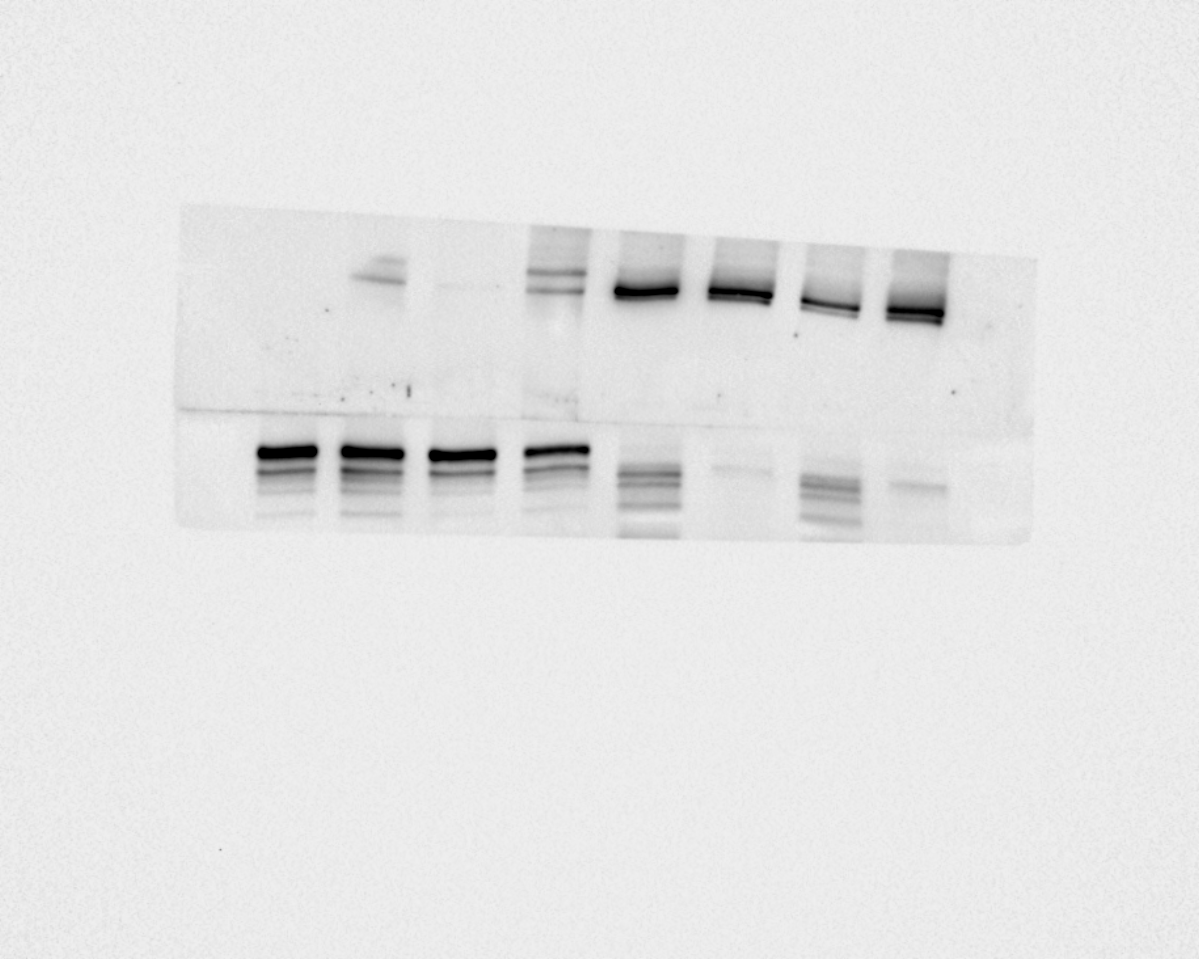

Supplement: Figure 7—source data 1. [file elife-70361-fig7-data1.zip › Figure 7-Source Data 1/Figure 7 full raw unedited/Figure 7C and D PlxD1 and Nrp1.tif]

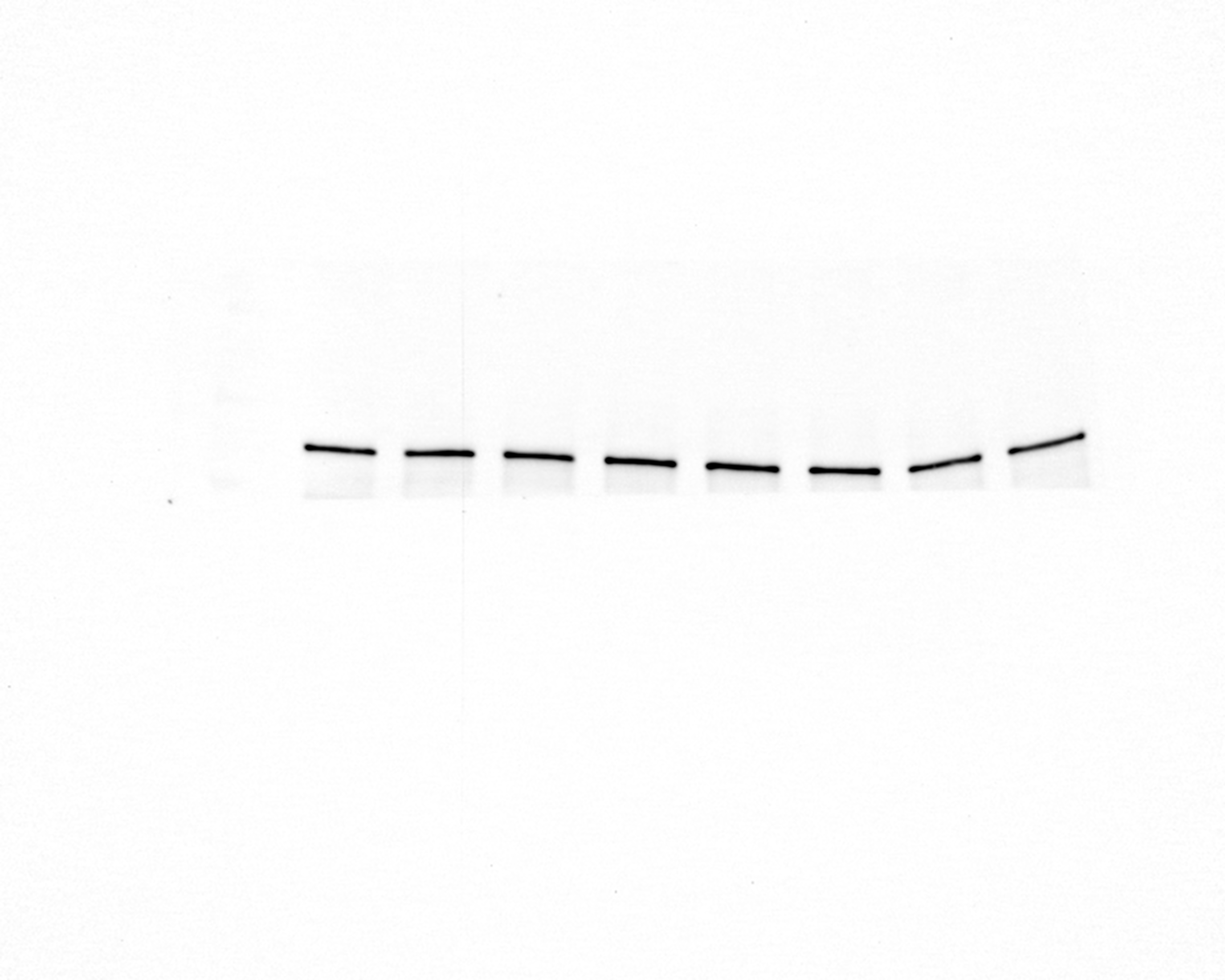

Supplement: Figure 9—source data 1. [file elife-70361-fig9-data1.zip › Figure 9-Source Data 1 /Figure 9 full raw unedited/Vinc de GSK3K-P.tif]

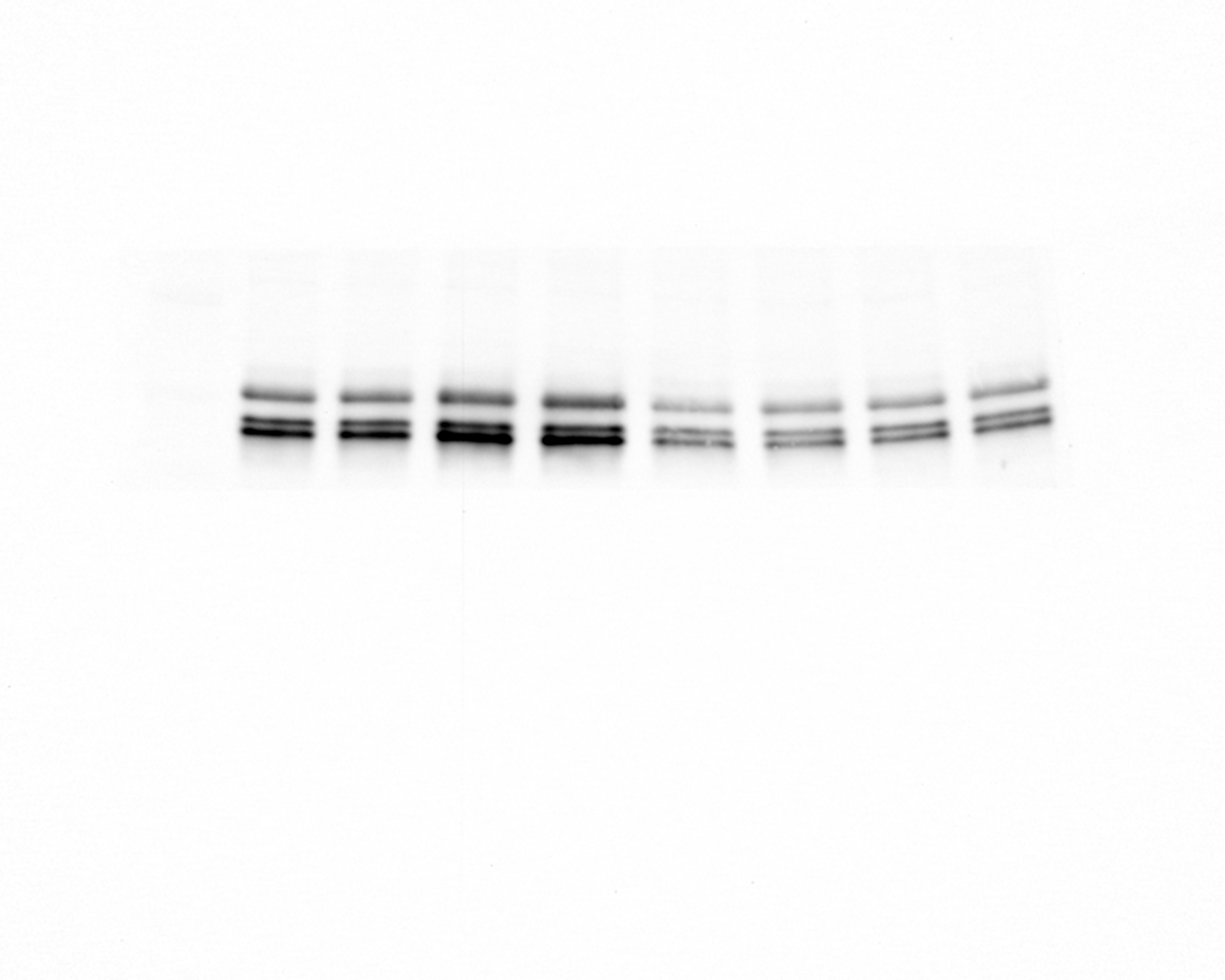

Supplement: Figure 9—source data 1. [file elife-70361-fig9-data1.zip › Figure 9-Source Data 1 /Figure 9 full raw unedited/GSK-P.tif]

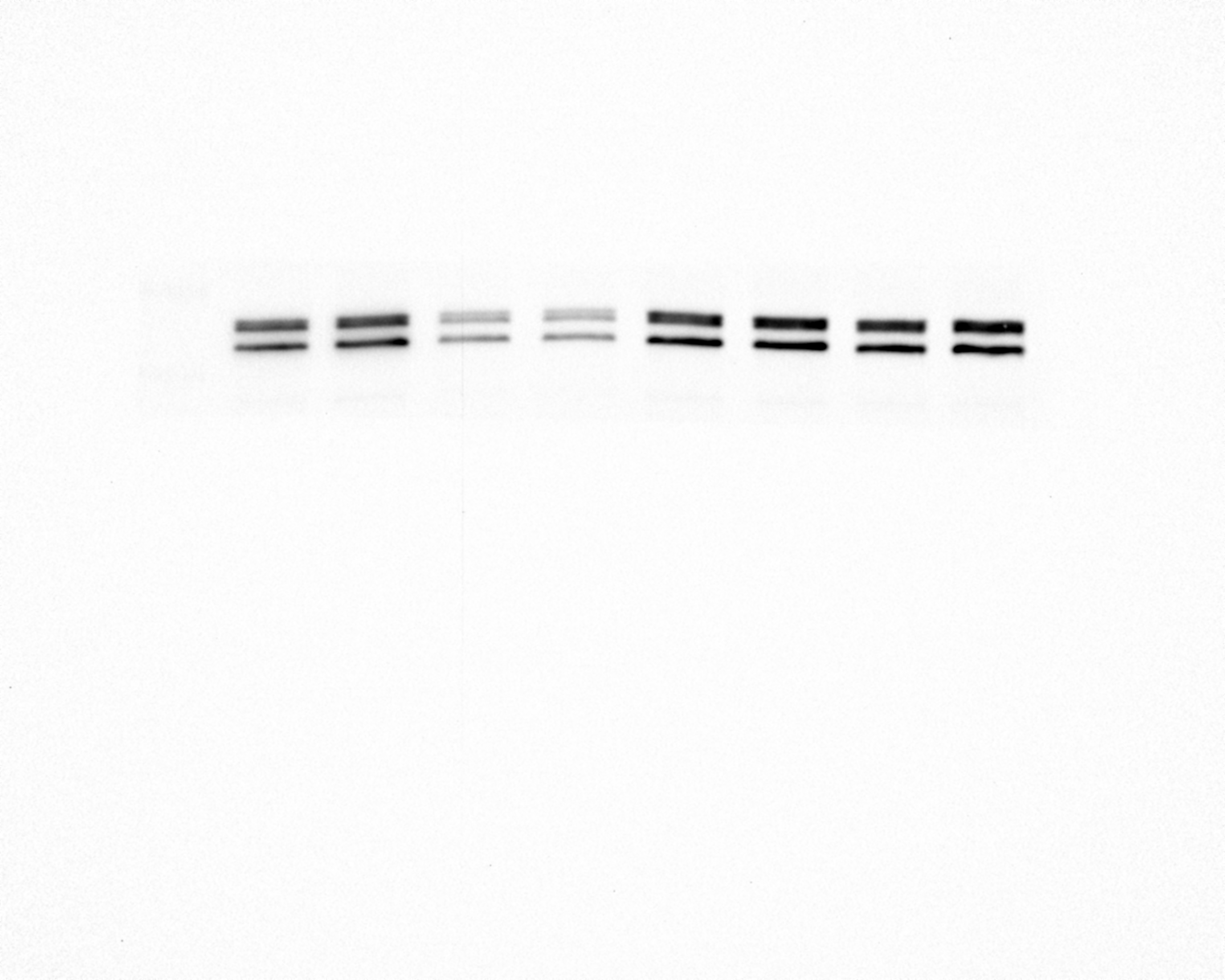

Supplement: Figure 9—source data 1. [file elife-70361-fig9-data1.zip › Figure 9-Source Data 1 /Figure 9 full raw unedited/CRMP4-P.tif]

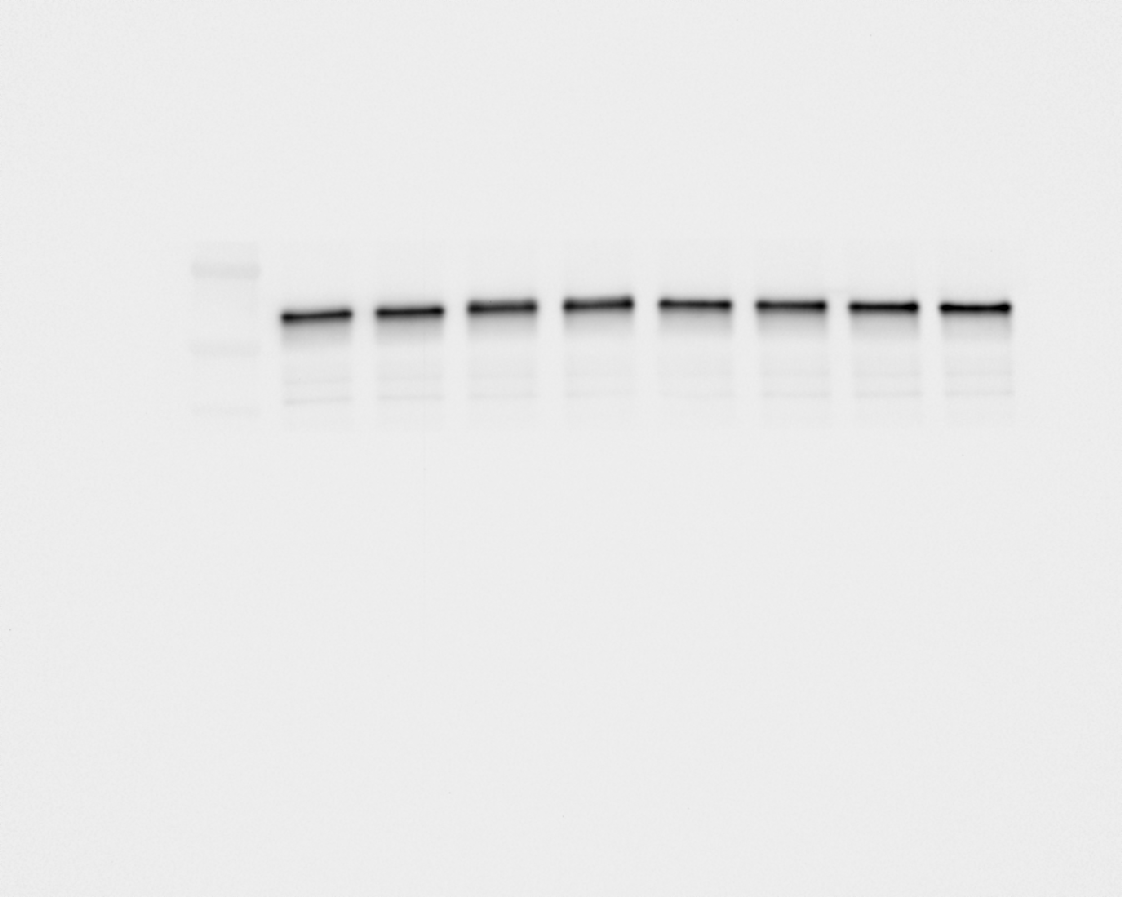

Supplement: Figure 9—source data 1. [file elife-70361-fig9-data1.zip › Figure 9-Source Data 1 /Figure 9 full raw unedited/AKT pan.tif]

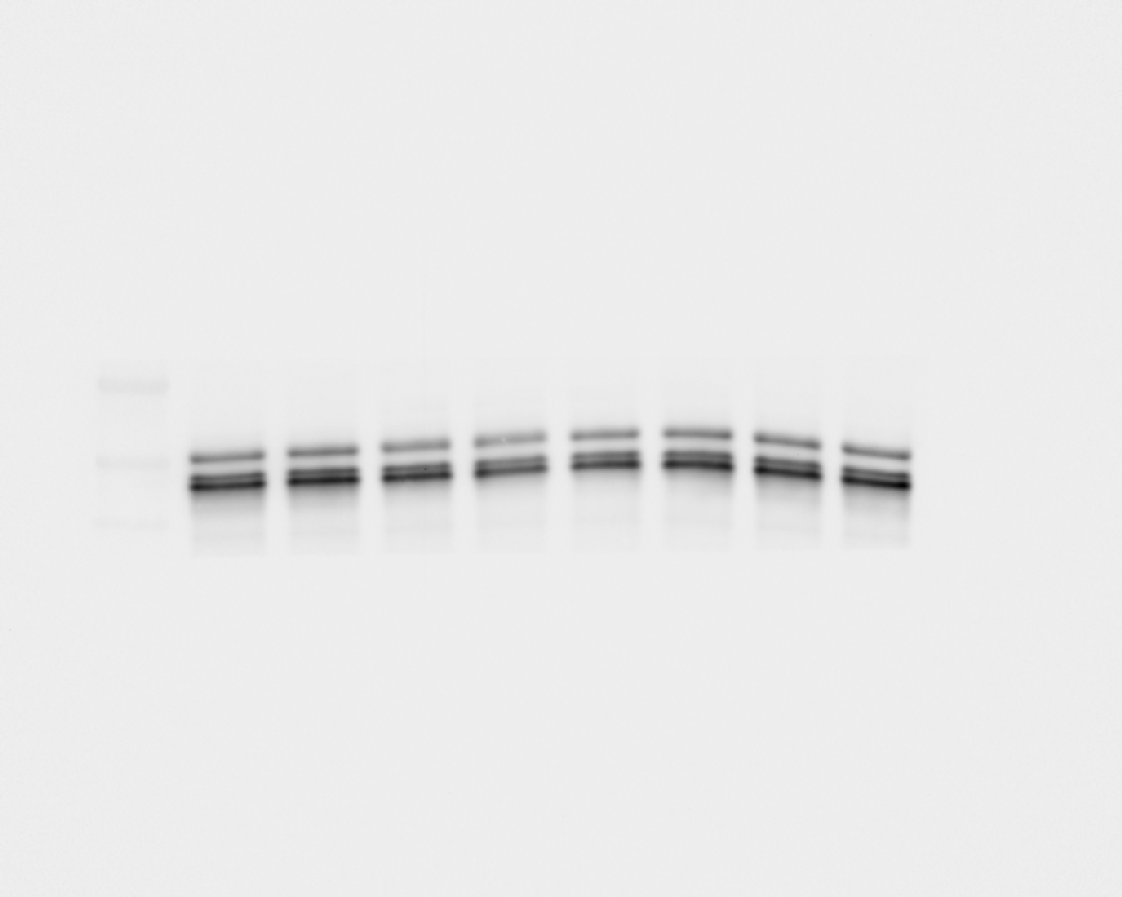

Supplement: Figure 9—source data 1. [file elife-70361-fig9-data1.zip › Figure 9-Source Data 1 /Figure 9 full raw unedited/GSK pan.tif]

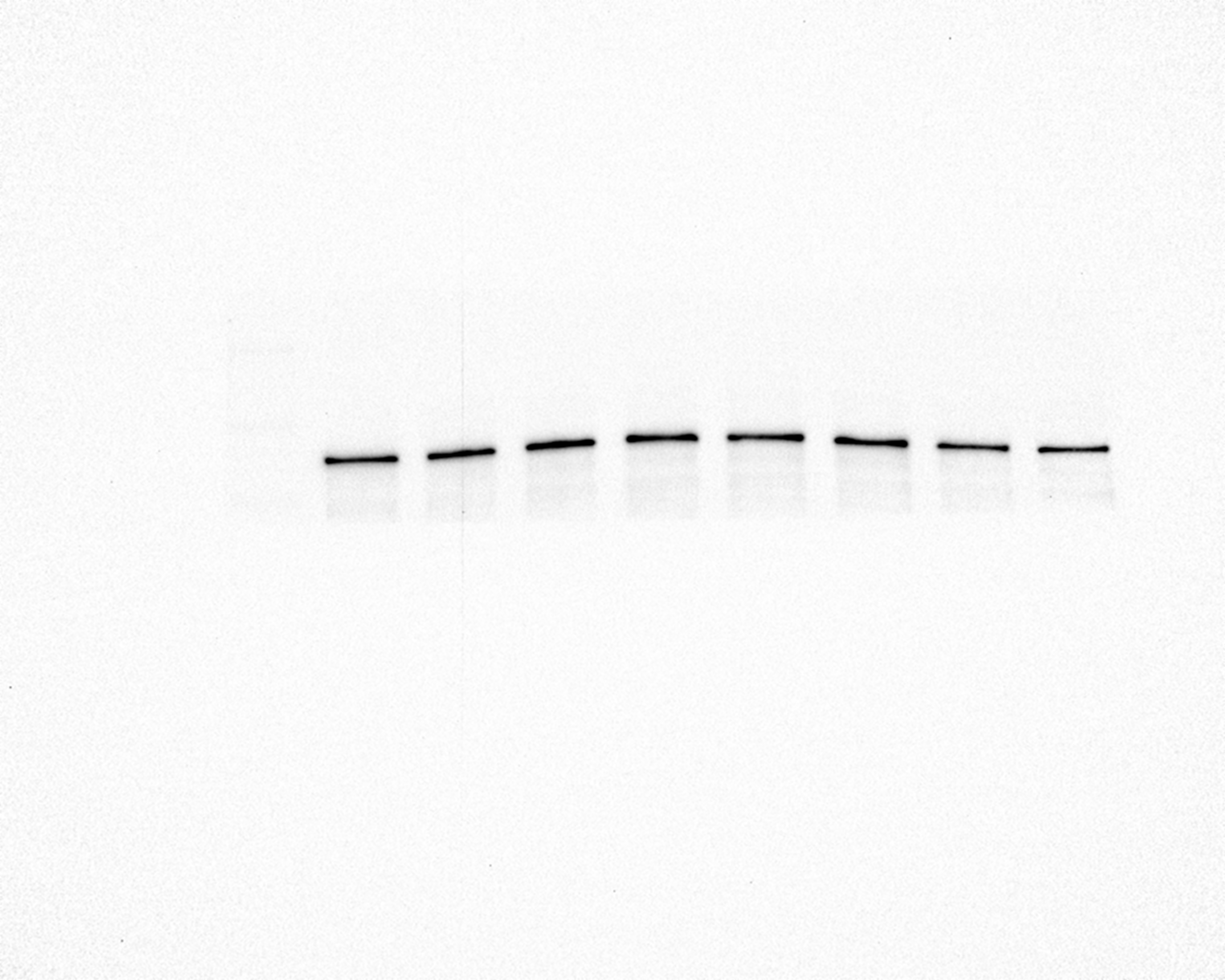

Supplement: Figure 9—source data 1. [file elife-70361-fig9-data1.zip › Figure 9-Source Data 1 /Figure 9 full raw unedited/vinc CRMP4-P.tif]

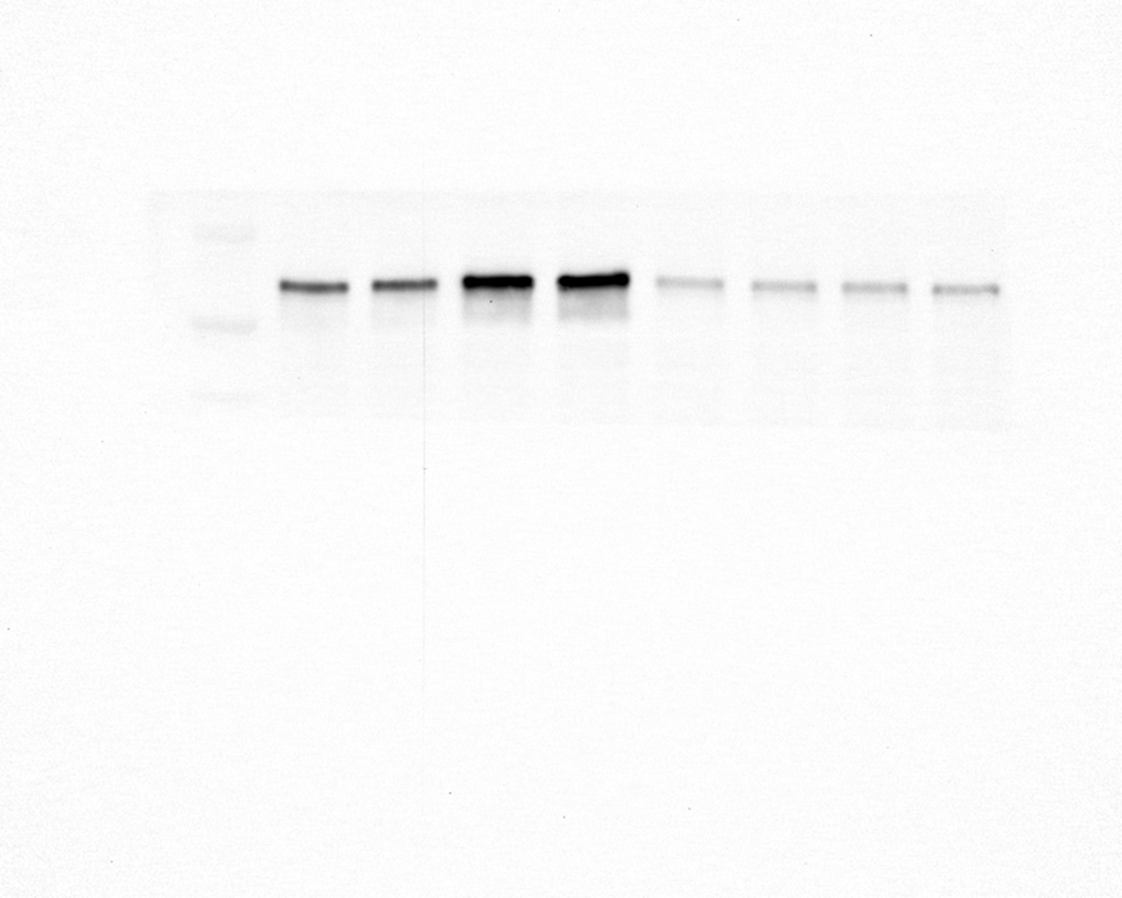

Supplement: Figure 9—source data 1. [file elife-70361-fig9-data1.zip › Figure 9-Source Data 1 /Figure 9 full raw unedited/AKT-P.tif]

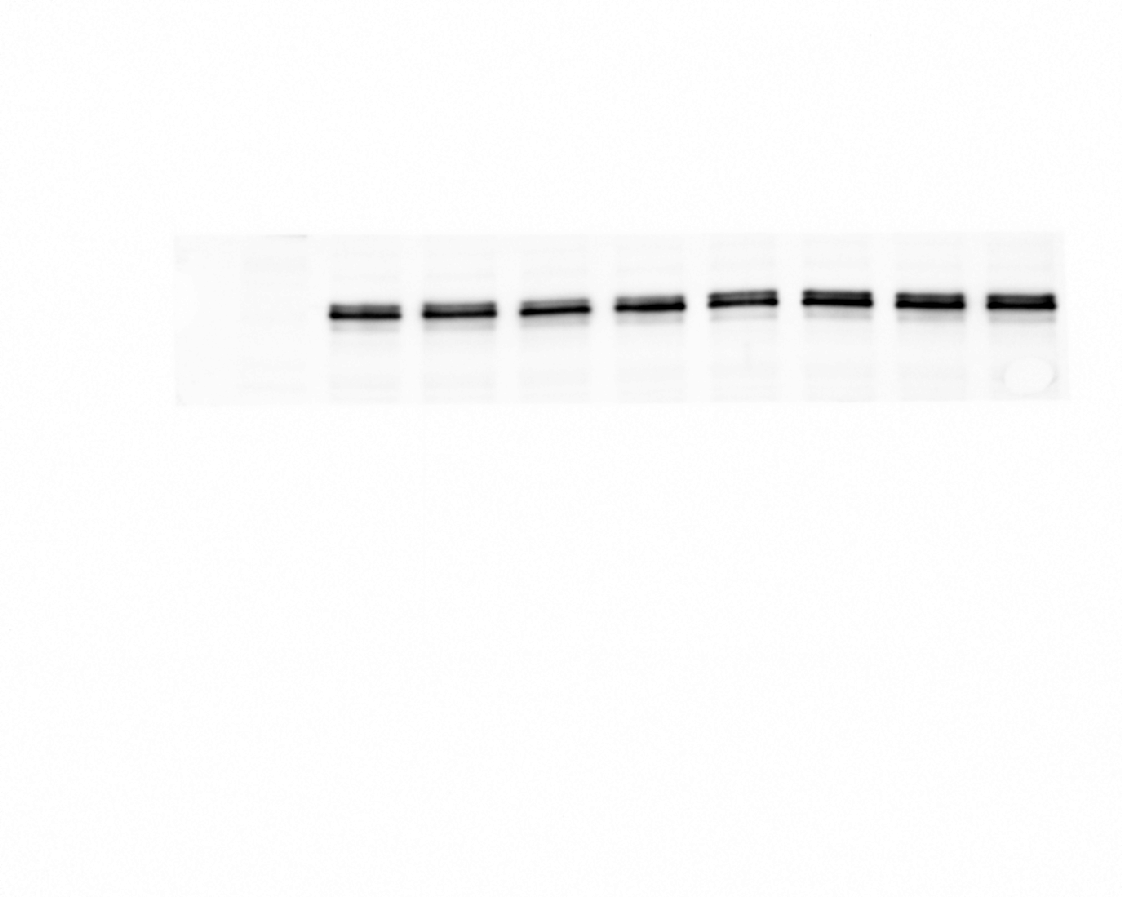

Supplement: Figure 9—source data 1. [file elife-70361-fig9-data1.zip › Figure 9-Source Data 1 /Figure 9 full raw unedited/TUC4.tif]

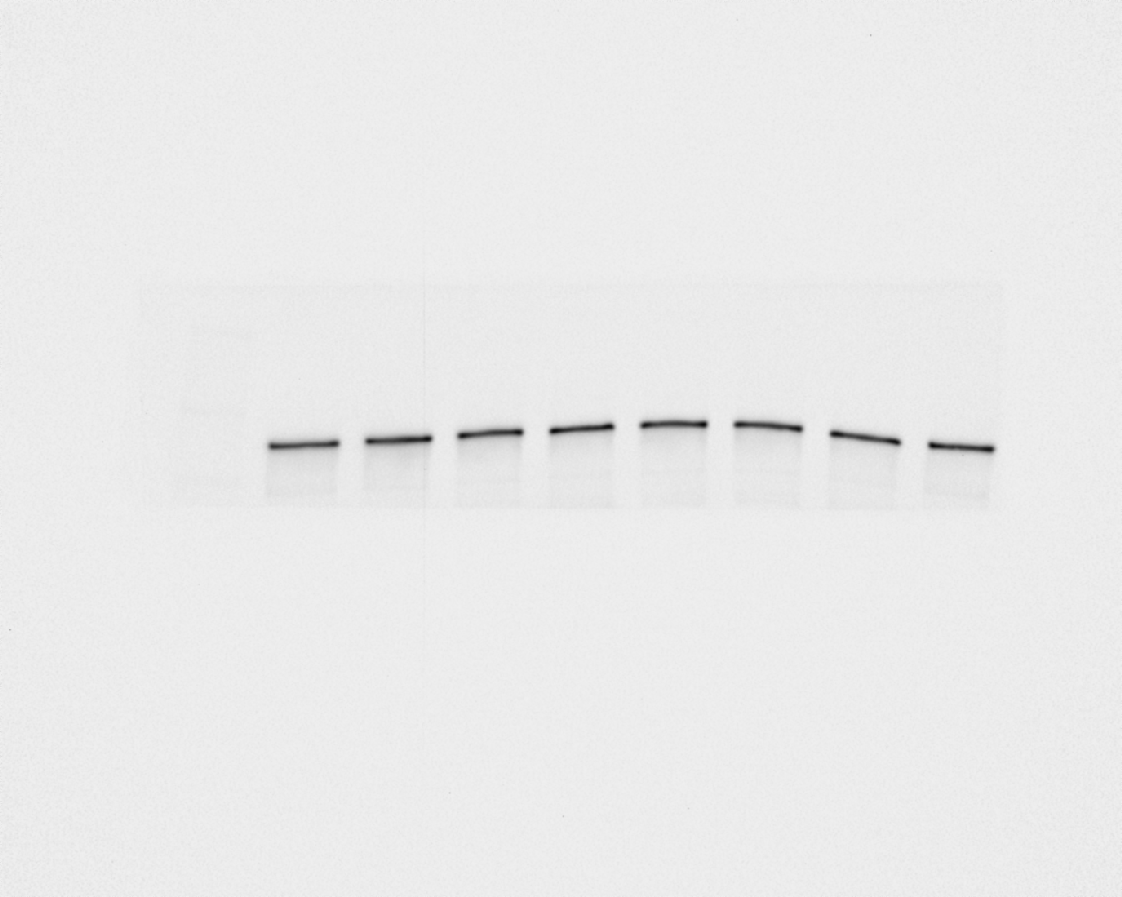

Supplement: Figure 9—source data 1. [file elife-70361-fig9-data1.zip › Figure 9-Source Data 1 /Figure 9 full raw unedited/Vinc de GSK pan.tif]

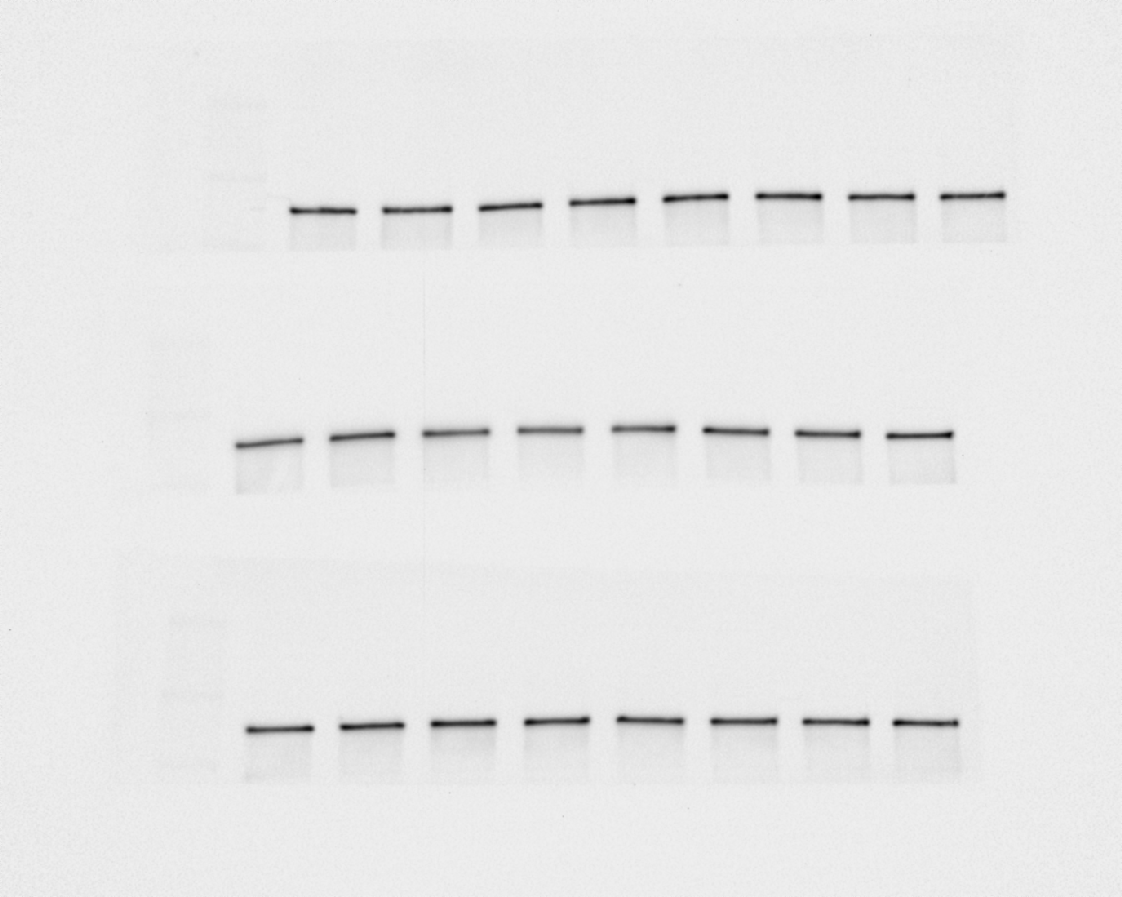

Supplement: Figure 9—source data 1. [file elife-70361-fig9-data1.zip › Figure 9-Source Data 1 /Figure 9 full raw unedited/Vinc TUC4 and AKT pan.tif]

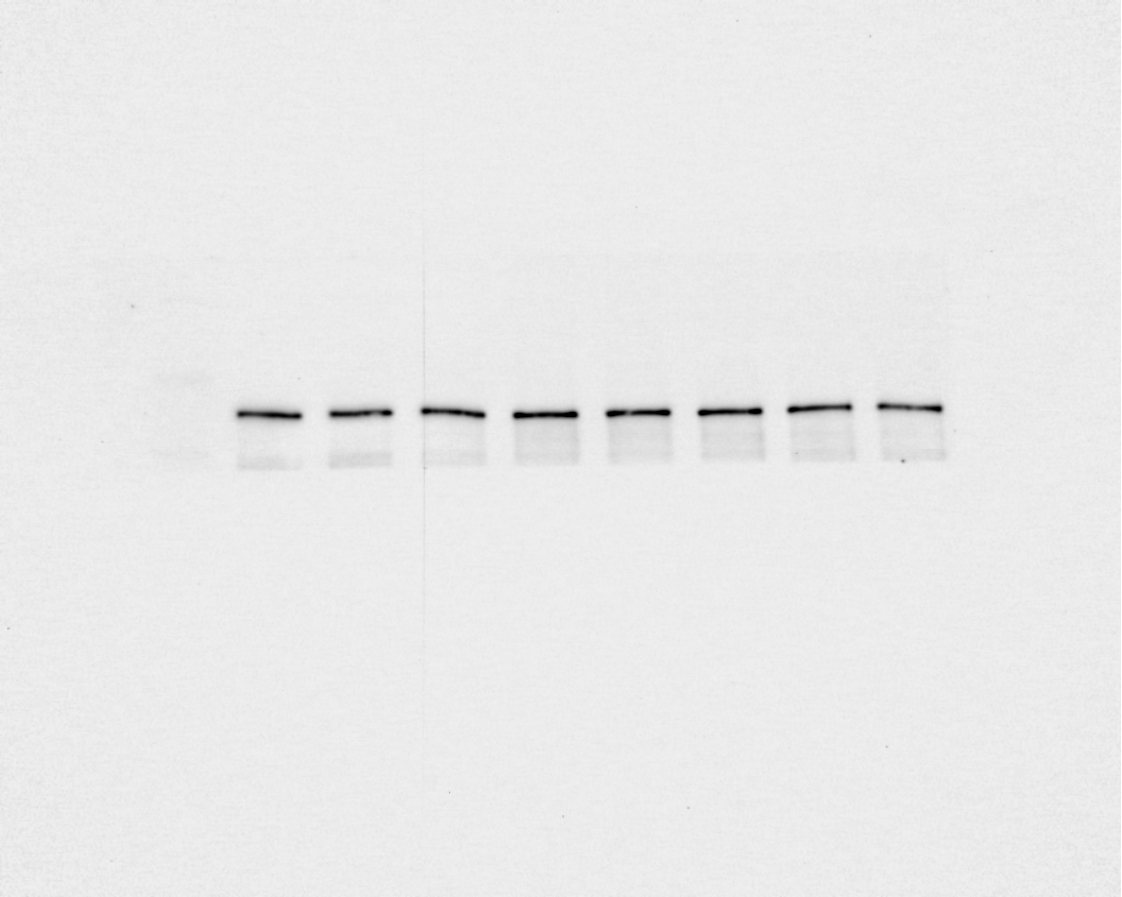

Supplement: Figure 9—source data 1. [file elife-70361-fig9-data1.zip › Figure 9-Source Data 1 /Figure 9 full raw unedited/Vinc AKT-P.tif]

Figure 10B

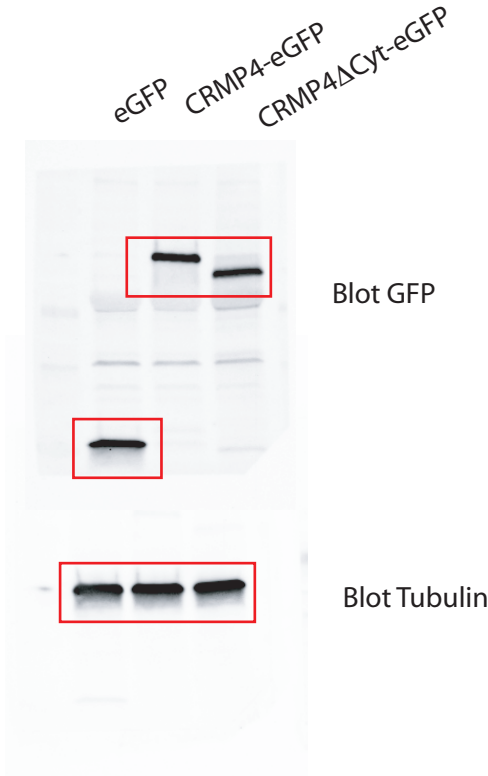

Figure 10C

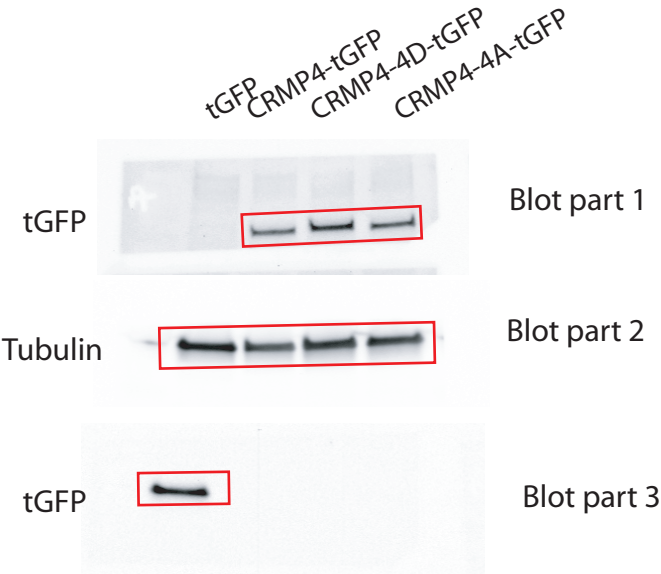

Supplement: Figure 10—source data 1. [file elife-70361-fig10-data1.zip › Figure 10-Source Data 1/Figure 10B and 10C uncropped blot and relevant bands.pdf]
