# Supplementary material for: CRMP4-mediated fornix development involves Semaphorin-3E signaling pathway
Source: eLife. 2021 Dec 3;10:e70361. doi: 10.7554/eLife.70361 (PMC8683083; doi:10.7554/eLife.70361)
Supplement: Figure 7—source data 1. [file elife-70361-fig7-data1.zip › Figure 7-Source Data 1/Figure 7 uncroppped blot with relevant bands .pdf]

Figure 7

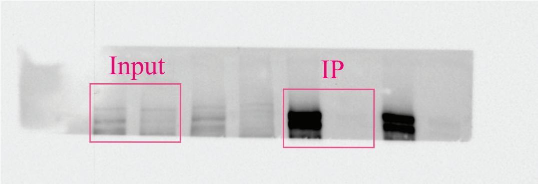

VEGFR2 (Fig 7A)

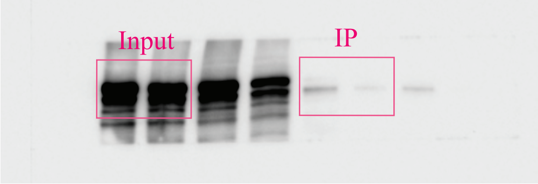

PlxD1 (Fig 7A)

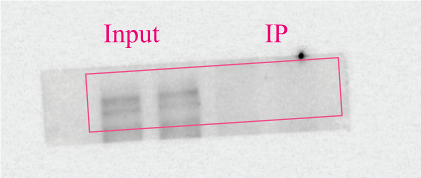

VEGFR2 (Fig 7B)

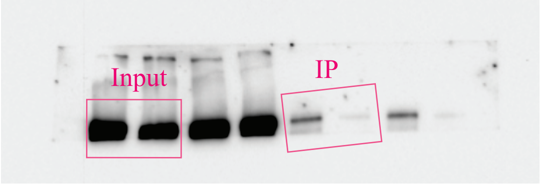

Nrp1 (Fig 7A)

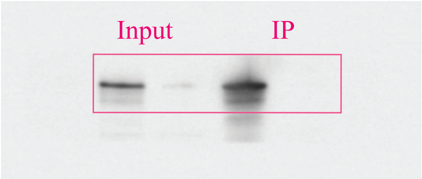

CRMP4 (Fig 7B)

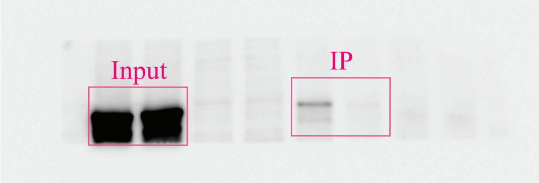

CRMP4 (Fig 7A)

Note that for PlxD1, the WB was revealed upside down

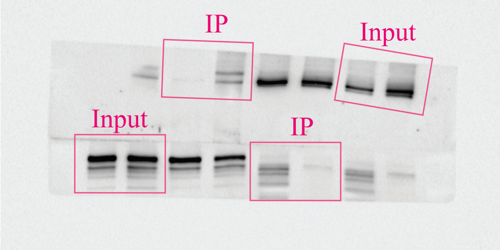

PlxD1 (Fig 7C)

Nrp1 (Fig 7D)

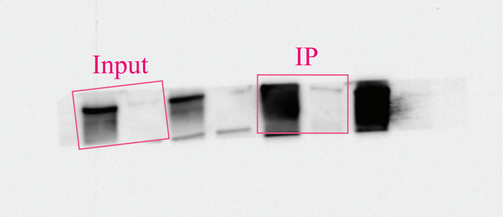

CRMP4
IP PlxD1 (Fig 7C)

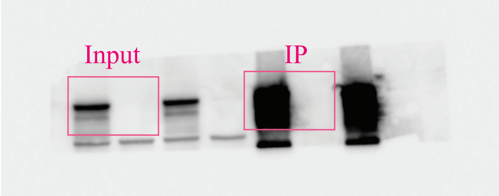

CRMP4
IP Nrp1 (Fig 7D)
