# Supplementary material for: CRMP4-mediated fornix development involves Semaphorin-3E signaling pathway
Source: eLife. 2021 Dec 3;10:e70361. doi: 10.7554/eLife.70361 (PMC8683083; doi:10.7554/eLife.70361)
Supplement: Figure 8—source data 1. [file elife-70361-fig8-data1.zip › Figure 8-Source Data 1/Figure 8 uncropped blot and relevant bands.pdf]

Figure 8

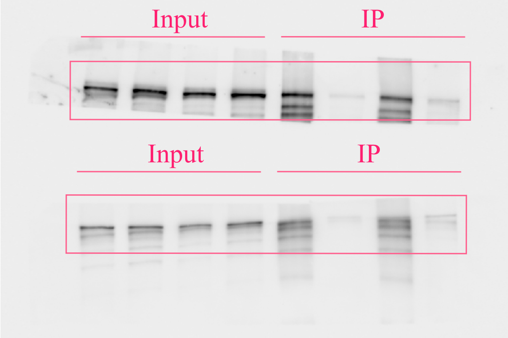

PlxD1 (Fig 8A)

Nrp1 (Fig 8A)

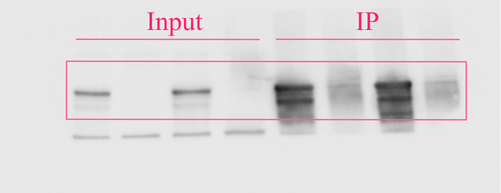

CRMP4 (Fig 8A)

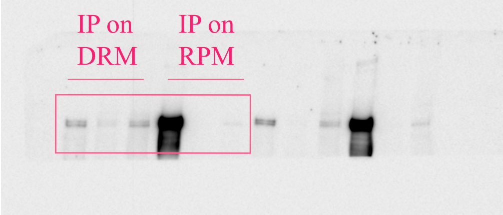

PlxD1 (Fig 8D)

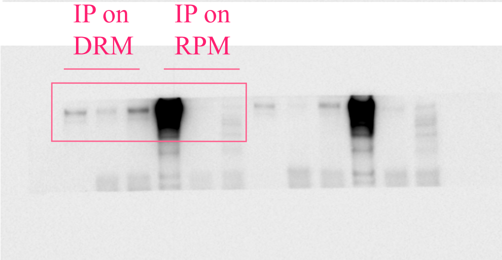

Nrp1 (Fig 8D)

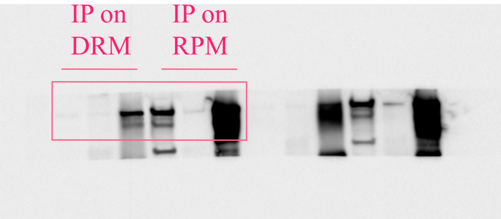

CRMP4 (Fig 8D)

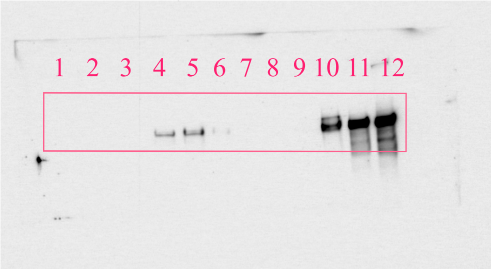

PlxD1 (Fig 8B)

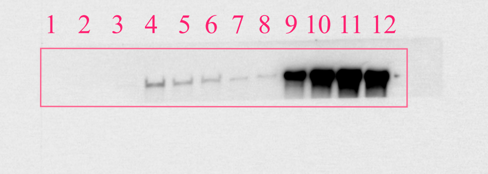

CRMP4 (Fig 8B)

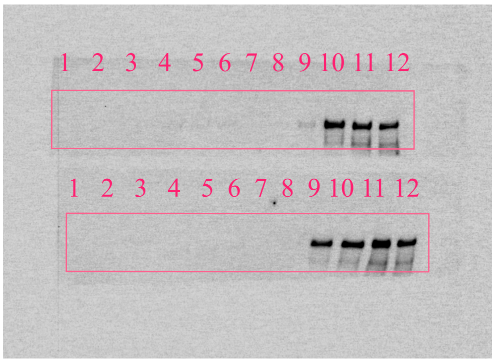

PlxD1(Fig 8C)

CRMP4 (Fig 8C)

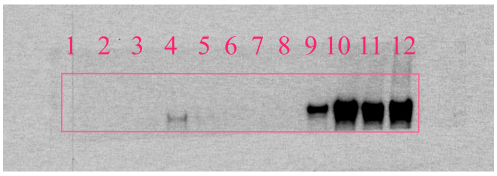

Nrp1 (Fig 8B)

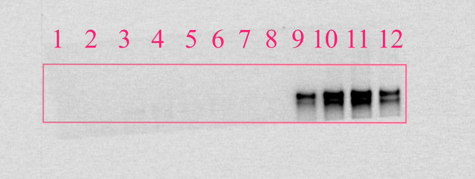

Nrp1 (Fig 8C)

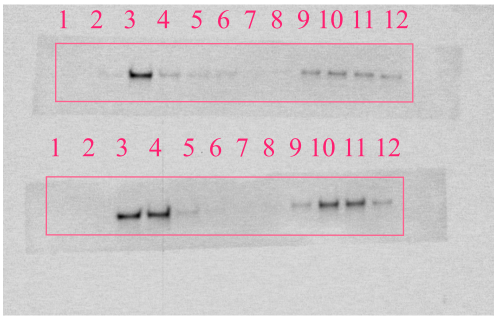

Flotilin (Fig 8C)

Flotilin (Fig 8B)
