# Supplementary material for: CRMP4-mediated fornix development involves Semaphorin-3E signaling pathway
Source: eLife. 2021 Dec 3;10:e70361. doi: 10.7554/eLife.70361 (PMC8683083; doi:10.7554/eLife.70361)
Supplement: Figure 9—source data 1. [file elife-70361-fig9-data1.zip › Figure 9-Source Data 1 /Figure 9 uncropped blot and relevant bands 2.pdf]

Figure 9C

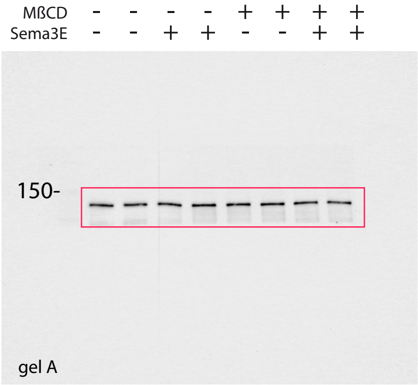

WB using mouse vinculin

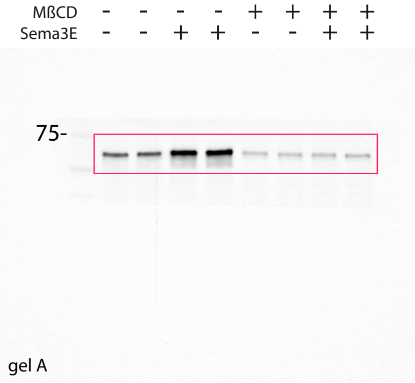

WB using rabbit phospho-AKT

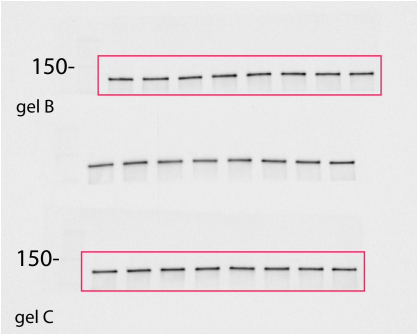

WB using mouse vinculin

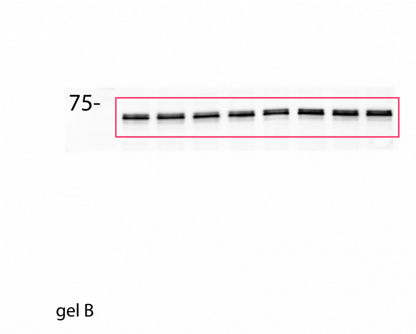

WB using rabbit CRMP4

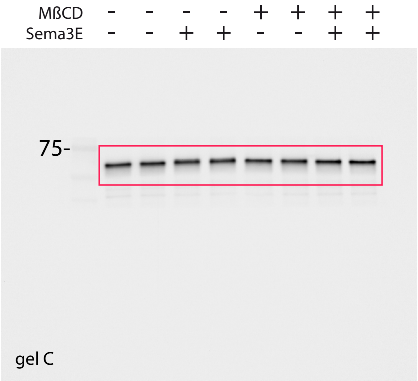

WB using rabbit AKT pan

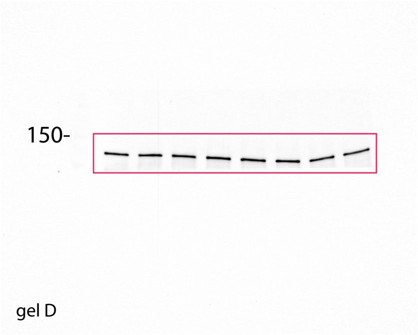

WB using mouse vinculin

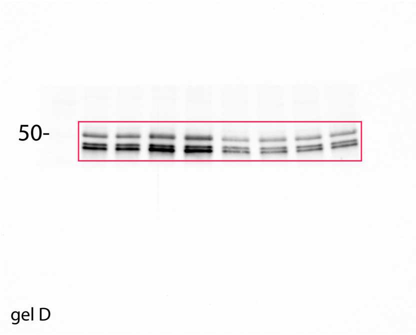

WB using rabbit phospo-GSK3 α/β

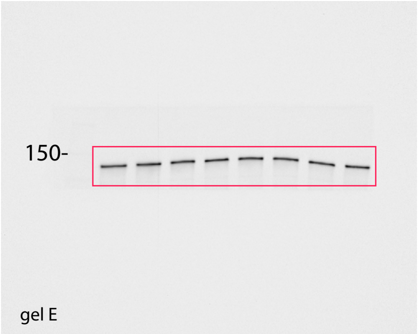

WB using mouse vinculin

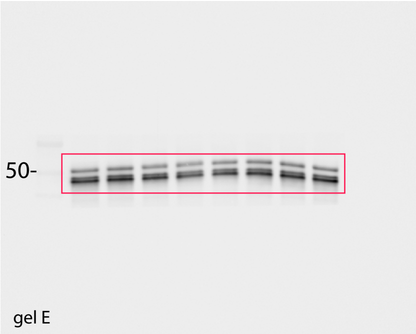

WB using rabbit GSK3 α/β pan

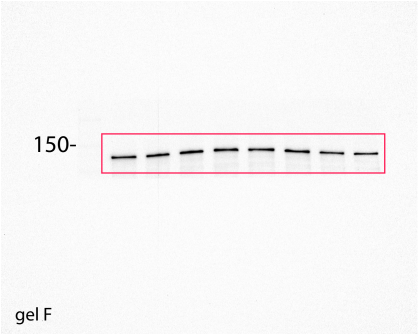

WB using mouse vinculin

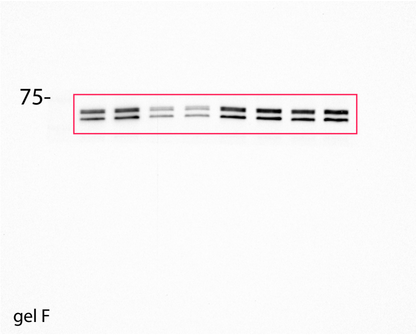

WB using rabbit phospo-CRMP4
